# Discovering Facet‐Dependent Formation Kinetics of Key Intermediates in Electrochemical Ammonia Oxidation by a Electrochemiluminescence Active Probe

**DOI:** 10.1002/advs.202402673

**Published:** 2024-06-23

**Authors:** Dina Sun, Jiaqi Zhang, Heng Wang, Yanxia Song, Jing Du, Genping Meng, Shihao Sun, Weihua Deng, Zhiyi Wang, Baodui Wang

**Affiliations:** ^1^ State Key Laboratory of Applied Organic Chemistry Key Laboratory of Nonferrous Metal Chemistry and Resources Utilization of Gansu Province Lanzhou University Lanzhou Gansu 730000 China; ^2^ School of Mathematics and Statistics Gansu Key Laboratory of Applied Mathematics and Complex Systems Lanzhou University Lanzhou 730000 China; ^3^ Spin‐X Institute School of Chemistry and Chemical Engineering State Key Laboratory of Luminescent Materials and Devices South China University of Technology Guangzhou 511442 China

**Keywords:** electrocatalytic AOR, electrochemiluminescence, facet, formation Kinetics, N_2_H_4_ intermediate

## Abstract

Facile evaluation of formation kinetics of key intermediate is crucial for a comprehensive understanding of electrochemical ammonia oxidation reaction (AOR) mechanisms and the design of efficient electrocatalysts. Currently, elucidating the formation kinetics of key intermediate associated with rate‐determining step is still challenging. Herein, 4‐phtalamide‐N‐(4′‐methylcoumarin) naphthalimide (CF) is developed as a molecular probe to detect N_2_H_4_ intermediate during AOR via electrochemiluminescence (ECL) and further investigated the formation kinetics of N_2_H_4_ on Pt catalysts with different crystal planes. CF probe can selectively react with N_2_H_4_ to release ECL substance luminol. Thus, N_2_H_4_ intermediate as a key intermediate can be sensitively and selectively detected by ECL during AOR. For the first time, Pt(100) facet is discovered to exhibit faster N_2_H_4_ formation kinetics than Pt(111) facet, which is further confirmed by Density functional theory calculation and the finite element simulation. The AOR mechanism under the framework of Gerischer and Mauerer is further validated by examining N_2_H_4_ formation kinetics during the dimerization process (NH_2_ coupling). The developed ECL active probe and the discovered facet‐dependent formation kinetics of key intermediates provide a promising new tool and strategy for the understanding of electrochemical AOR mechanisms and the design of efficient electrocatalysts.

## Graphical abstract

4‐phtalamide‐N‐(4'‐methylcoumarin) naphthalimide (CF) is utilized as a molecular probe for the detection of N_2_H_4_ to directly investigate the formation kinetics of N_2_H_4_ intermediate on Pt catalysts with different crystal planes during the electrocatalytic ammonia oxidation reaction (AOR) by time‐dependent electrochemiluminescence (ECL). N_2_H_4_ is confirmed to be a key intermediate in electrocatalytic AOR and Pt(100) facet demonstrated faster N_2_H_4_ formation kinetics than Pt(111) facet.

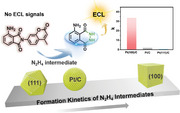

## 1 Introduction

The electrocatalytic ammonia oxidation reaction (AOR) catalyzed by transition metal catalysts has been found many industrial and engineering applications, such as ammonia fuel cells, hydrogen production with ammonia electrolyzers, wastewater treatment and nitrogen cycles.^[^ ^1^, ^2^, ^3^, ^4^, ^5^, ^6^ ^]^ However, its kinetic limitations accounts for a lager overpotential required for ammonia electrolysis, resulting in the considerable energy penalty in the electrode.^[^ ^7^ ^]^ The in‐depth study of the electrochemical mechanism of AOR is crucial for the rational design of efficient AOR electrocatalysts. Currently, two mechanisms are generally accepted for this process, namely Oswin–Salomon (O–S) and Gerischer–Mauerer (G–M) mechanism.^[^ ^8^ ^]^ The G–M mechanism is favored by many theoretical calculations or experimental studies due to its low onset potential.^[^ ^9^, ^10^, ^11^ ^]^ According to the G–M mechanism, the N_2_H_x+y_ intermediates generated from NH_x_ and NH_y_ (x, y = 1 or 2) coupling constitutes the pivotal precursor to the generation of N_2_.^[^ ^12^ ^]^ A previous study suggested that NH_2_ fragment coupling should be the rate‐determining step at the interface between solid and liquid.^[^ ^13^ ^]^ In consequence, the detection of N_2_H_x+y_ intermediate species and study their formation rate on the electrode surface are very critical to understand the AOR mechanism and electrochemical kinetics.

Generally, research on the mechanisms of electrocatalytic AOR on the metal catalysts surface has always been a focus of attention. As a result of complexity of the internal mechanism, the AOR reaction kinetics is not only influenced by the catalyst types, but also by a variety of structural patterns.^[^ ^8^ ^]^ Recent efforts have been dedicated to enhance the electrocatalytic AOR efficiency of Pt‐based catalyst. Pt(100) planes have been found to demonstrate superior performance than Pt(111) planes in many cases.^[^ ^14^, ^15^, ^16^ ^]^ Bertin et al. explored the catalytic activity of Pt nanoparticles (NPs) with preferential orientation (100) planes and polycrystalline Pt catalysts on AOR, indicating that Pt NPs with (100) planes have higher efficiency than polycrystalline Pt.^[^ ^17^ ^]^ DFT calculations showed that the easier formation of ^*^NH_2_ on the crystal surface of Pt(100) leads to a lower dimerization barrier of ^*^N_2_H_4_, which is the reason for the higher ammonia oxidation rate on Pt(100) planes.^[^ ^8^ ^]^ Although the active difference of Pt(100) and Pt(111) planes is already significant, the fundamental reason and internal mechanism of structure sensitivity of Pt facets on AOR is highly controversial. Accordingly, the evaluation of dimerization kinetics of ^*^NH_2_ during electrocatalytic AOR on different Pt crystal planes play indispensable roles in the deeper understanding of mechanisms of electrocatalytic AOR. However, there is a lack of important experimental evidence for the study of the dimerization product (N_2_H_4_) on different Pt crystal planes.

In recent years, several techniques have been employed for the detection of intermediates, including in situ Fourier transform infrared spectroscopy (FTIR),^[^ ^18^, ^19^, ^20^ ^]^ surface enhanced Raman spectroscopy,^[^ ^21^, ^22^, ^23^ ^]^ differential electrochemical mass spectroscopy (DEMS),^[^ ^24^, ^25^, ^26^, ^27^ ^]^ and rotating ring‐disk electrodes.^[^ ^28^, ^29^, ^30^ ^]^ Even though these techniques have the impressive amount of information on intermediate detection, they suffer from their own intrinsic limitations. For example, the quantitative analysis provided by FTIR has low sensitivity and significant errors, which hinder its accuracy; in situ Raman analysis usually requires specific experimental conditions, which may have an impact on the sample; DEMS faces challenges in detecting certain anti volatile liquids and ion products. Most importantly, since the electrochemical AOR process involves multi‐step dehydrogenation and coupling reactions,^[^ ^8^ ^]^ a technique for the analysis of the formation rate of intermediate species in the speed determination step is critically important in understanding real kinetics and sophisticated correlations between structure and function. Especially, the study of formation dynamics of N_2_H_4_ generated in the rate‐determining step is still lacking, limiting the in‐depth understanding of AOR mechanisms. Therefore, there is an urgent need to find simpler and more efficient techniques for the quantitative observation of N_2_H_4_ during electrochemical reaction to fully understand the AOR mechanism and advance our knowledge for optimizing electrocatalysts.

As a powerful tool for molecular identification, electrochemiluminescence (ECL) is a specific luminescence reaction triggered by electrochemistry on the electrode surface, involving electrochemistry and chemiluminescence.^[^ ^31^, ^32^, ^33^ ^]^ Due to its versatility, near‐zero background signal, high sensitivity and wide dynamic range, ECL technology has made significant progress in various research fields such as environmental and food analysis,^[^ ^34^, ^35^, ^36^ ^]^ immunoassays,^[^ ^37^, ^38^, ^39^ ^]^ pharmaceutical and nucleic acid hybridization analysis.^[^ ^40^, ^41^, ^42^ ^]^ Keeping in this mind, the exceedingly promising potential of ECL time‐resolved spectroscopy for monitoring the intermediate species during electrochemical processes cannot be underestimated. However, so far, due to the absence of probes with good ECL activity and highly selective responses to intermediates, monitoring the intermediate species with ECL spectrum during AOR remains challenging.

Herein, inspired by the use of molecular probes in catalytic research, 4‐phtalamide‐N‐(4′‐methylcoumarin) naphthalimide (CF) as the molecular probe has been synthesized whose ECL signal can respond to N_2_H_4_ with high sensitivity and specificity. Upon a Gabrieltype hydrazinolysis of the CF probe in the presence of N_2_H_4_, the probe will be cleaved into ECL molecule luminol and 7‐amino‐4‐methylcoumarin (AMC) with a high fluorescence quantum yield.^[^ ^43^ ^]^ With this probe, N_2_H_4_ intermediates can be monitored in Pt with different crystal planes catalyzed electrochemical AOR by using time‐dependent ECL technology. The analysis of N_2_H_4_ generation kinetics shows that Pt(100) crystal planes have a higher N_2_H_4_ intermediate generation rate than the Pt(111) crystal planes, which is in good agreement with DFT calculation results. The finite element simulation (FES) further confirms the sensitivity of the crystal plane structure of N_2_H_4_ intermediate evolution. Both experimental and computational results reveal the deep mechanism of performance differences between Pt(100) and Pt(111) crystal planes: the generation kinetics of N_2_H_4_ intermediates on Pt(100) crystal planes is significantly superior to Pt(111) crystal planes. The developed ECL active probe offers a powerful new tool for both the quantitative detection of N_2_H_4_ intermediates and the discovery of facet‐dependent formation kinetics of N_2_H_4_ intermediates during electrocatalytic AOR, helping the understanding of electrochemical AOR mechanisms and the design of efficient electrocatalysts.

## 2 Results and Discussion

**Figure 1:**
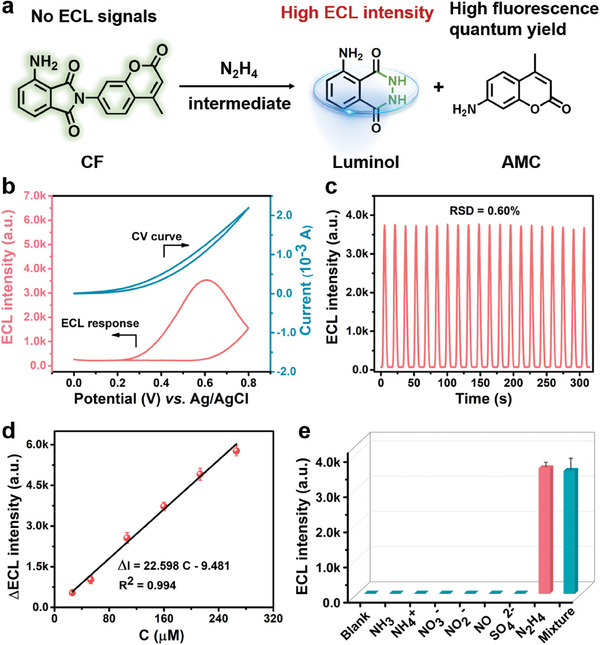
a) Schematic illustration of the Gabrieltype hydrazinolysis reaction of the CF probe in the presence of N_2_H_4_. b) ECL response of CF to N_2_H_4_ at bare GCE in 10 mm PBS (pH = 7.4) containing 0.05 mm H_2_O_2_. c) Stability test of the ECL response of CF to N_2_H_4_ under 20 repetitive cyclic potential scans (voltage of PMT, 800 V). d) Linear relationship between the concentration of N_2_H_4_ and ECL intensity in 10 mm PBS (pH = 7.4) containing 0.05 mm H_2_O_2_. e) Selectivity of ECL response to intermediate detection during AOR process.

The selection of ECL active probe is vitally important for the detection of intermediates. CF was successfully synthesized in this study as the molecular probes of specific response to N_2_H_4_ intermediates (Figures S1–S5, Supporting Information). First, the probe should be able to react with N_2_H_4_ intermediates with high specificity and exhibit excellent ECL properties. Additionally, ECL response of the probe to N_2_H_4_ should exhibit the strong regularity within a certain range to achieve quantitative evaluation of N_2_H_4_ intermediates during electrocatalytic AOR. Accordingly, we validated these requirements in **Figure** **1**. Upon a Gabrieltype hydrazinolysis of the CF probe in the presence of N_2_H_4_, the probe is cleaved into ECL molecule luminol and 7‐amino‐4‐methylcoumarin (AMC) (Figure 1a). As shown in Figure 1b, the response of CF to N_2_H_4_ exhibits an obvious ECL signal at 0.6 V (vsAg/AgCl) at bare GCE in 10 mm PBS (pH = 7.4) containing 0.05 mm H_2_O_2_, and there is no ECL signals in the absence of N_2_H_4_ (Figure S6, Supporting Information). Moreover, CF showed a stable ECL signal when the electrode was scanned in the potential range of 0–1.0 V, exhibiting ultra‐low relative standard deviation (RSD = 0.60%) (Figure 1c). Figure 1d and Figure S7 (Supporting Information) reveals the relationship between ECL intensity and concentrations of N_2_H_4_ in the range of 0–250 µm, indicating that there is a good positive correlation linear relation between ECL intensities of the sensor and concentrations of N_2_H_4_. The limit of detection (LOD) is estimated as low as 45 nm (signal/noise = 3.3).

Additionally, some possible species generated during the electrocatalytic AOR process may cause ECL signal interference. The selective experiment indicates that CF itself has no ECL response in the absence of N_2_H_4_, illustrating that CF molecule has no contribution to ECL signal change during AOR. Clearly, the reaction between CF and N_2_H_4_ produced strong ECL, whereas pretreatment with other competing compounds (excessive) did not cause generation of any ECL signals (Figure 1e). It is generally known that N_2_H_2_ is unstable and readily reacts with itself to quickly form N_2_H_4_ and N_2_ gas.^[^ ^44^ ^]^ In addition, CF probe only reacts specifically with N_2_H_4_ to generate a luminol luminophore.^[^ ^43^ ^]^ Therefore, the effect of N_2_H_2_ on ECL intensity of probe is minimal. Briefly, the ECL response of CF to N_2_H_4_ exhibits high selectivity in the electrochemical AOR process. Consequently, the probe CF is expected to be used for recognition of N_2_H_4_ in AOR process.

**Figure 2:**
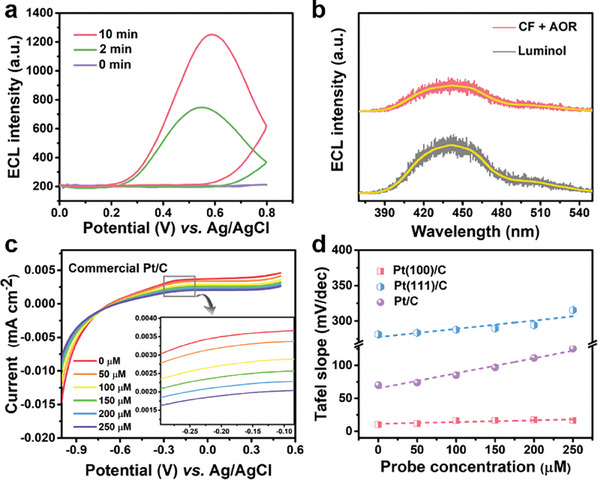
a) ECL response of CF after reaction with N_2_H_4_ generated by AOR with different accumulated time. b) ECL spectral comparison of N_2_H_4_ intermediates reacted with CF and luminol molecules. c) Linear sweep voltammetry curves of the commercial Pt/C electrocatalyst in 0.1 m KOH with the titration of 0–250 µm probes. Inset: zoom‐in of the gray boxed region in the main panel. d) The calculated Tafel slopes over catalysts of commercial Pt/C, Pt(100)/C, and Pt(111)/C with different exposed crystal faces under different CF concentrations.

To explore whether the reactive probe CF can be applied to the detection of N_2_H_4_ intermediates during electrocatalytic AOR, three prerequisites were proposed for ECL to be applicative in AOR kinetics evaluation: 1) whether N_2_H_4_ was produced in electrocatalytic AOR; 2) could N_2_H_4_ trigger ECL signals of CF; 3) would N_2_H_4_ generated during AOR be play a significant role in AOR kinetics. Our previous experiments have indubitably indicated that CF has a specific ECL response to N_2_H_4_. Therefore, CF is directly used as a specific probe for N_2_H_4_ intermediates to investigate the presence of N_2_H_4_ in the AOR process. Taking commercial Pt/C electrocatalyst as an example, clear ECL signals can be observed after driving AOR at a potential window of −1 to 0.5 V (vs Ag/AgCl) for a period of time, and the ECL intensity increases upon extended reaction time from 2 to 10 min (**Figure** **2**a). In addition, ECL signals generated by the reaction between CF and AOR intermediates can be recorded at 440 nm, which is consistent with the luminescence wavelength position of luminol (Figure 2b),^[^ ^45^ ^]^ further illustrating that the luminol produced by the hydrazinolysis reaction between CF and the N_2_H_4_ intermediate is the source of ECL signals rather than other cracking products.

In order to more accurately reflect the current density of three Pt‐based catalysts, the electrochemical active surface area (ECSA) was estimated by measuring the capacitive current associated with double‐layer charging from the scan‐rate dependence of cyclic voltammograms (CVs). The ECSA of Pt(100)/C, commercial Pt/C and Pt(111)/C is 17.5, 9.0, and 9.8, respectively (Figure S8, Supporting Information). In the following electrochemical experiments, ECSA was further used to normalize current density. Figure 2c represents linear sweep voltammetry (LSV) curves of the commercial Pt/C electrocatalyst in 0.1 m KOH with the titration of 0–250 µm probes, indicating that the titration of CF gradually reduces AOR current in a concentration‐dependent manner. Also, AOR current still presented when the probe concentration increased to 250 µm, which eliminated the possibility of CF probes poisoning the Pt/C surface.

Tafel analysis is an effective electrochemical tool for studying the reaction pathways of electrocatalytic processes. Generally, the smaller the Tafel slope value, the faster the current density increases, indicating faster catalyst kinetics and better catalytic activity.^[^ ^46^ ^]^ According to Tafel curve of commercial Pt/C catalysts, it can be seen that Tafel slope shows an increasing trend with the accumulation of CF concentration, indicating the change of reaction kinetics after the introduction of the probe (Figure S9, Supporting Information). Similarly, the Tafel slope of Pt(100)/C and Pt(111)/C catalysts exhibits a similar trend to commercial Pt/C catalysts, further indicating that the N_2_H_4_ intermediate has been successfully captured by CF (Figure S10, Supporting Information). Figure 2d compares the effect of CF titration on three catalysts, among which Pt(100)/C catalyst demonstrates the lower Tafel slope than Pt(111)/C catalyst, indicating that Pt(100) has faster AOR reaction kinetics than Pt(111). The Tafel slope of all three catalysts increases linearly with the increasing concentration of CF probe, illustrating the reduction of reaction kinetics of electrocatalytic AOR. These results further demonstrate the vital role of N_2_H_4_ intermediates in the reaction kinetics during the electrocatalytic AOR process.

**Figure 3:**
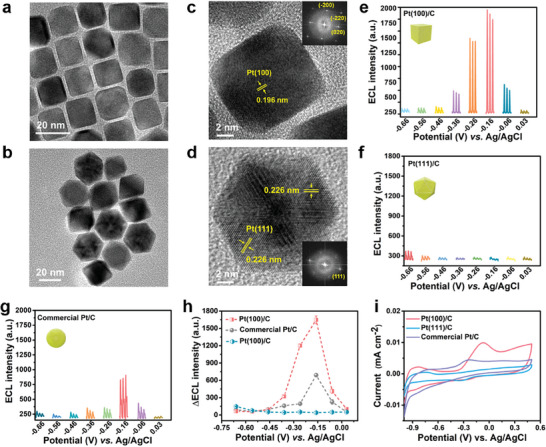
a) Typical TEM image of Pt cube and b) Pt icosahedron. c) HRTEM and Fourier transform (FFT) images of Pt cube and d) Pt icosahedron. ECL response of CF after reaction with N_2_H_4_ generated intermediates on e) Pt(100)/C, f) Pt(111)/C, and g) commercial Pt/C catalysts at different potentials. h) ECL intensity comparison of three catalysts with background correction. i) CV curves of commercial Pt/C, Pt(100)/C, and Pt(111)/C.

To verify the feasibility and repeatability of the established method for detecting intermediates during AOR, the commercial Pt/C (20%) was selected as the model electrocatalyst in this work, which has been shown to be an efficient electrocatalyst in the AOR process according to previous reports.^[^ ^47^ ^]^ In addition, shape‐controlled Pt NPs was synthesized and deposited onto Vulcan carbon XC‐72R according to the previously reported method to study the effect of crystal face on AOR behavior.^[^ ^48^ ^]^ Figure S11 (Supporting Information) shows X‐ray diffraction (XRD) patterns of the commercial Pt/C, Pt(100)/C, and Pt(111)/C. In all diffractograms, characteristic peaks of the face centered cubic structure (FCC) of Pt appear at 39.8°, 46.2°, 67.5°, and 81.3°, which is associated with the crystal faces of (111), (200), (220), and (311), respectively.^[^ ^49^ ^]^ All Pt shape‐controlled NPs exhibited well‐defined shapes and structures. Figure S12 (Supporting Information) and **Figure** **3**a,b depicts transmission electron microscopy (TEM) images of shape‐controlled Pt NPs. The average size of the Pt cube and the Pt icosahedron is 10.7 and 18.3 nm, respectively. Also, the crystal faces of Pt(100) and Pt(111) are verified by high‐resolution transmission electron microscopy (HRTEM) and Fourier transform (FFT) images. Clearly, the lattice spacing of 0.226 nm (Pt icosahedron) and 0.196 nm (Pt nanocube) are in good agreement with Pt(111) and Pt(100) crystal planes, respectively (Figure 3c,d). It is noted that commercial Pt/C, Pt(100)/C and Pt(111)/C all exhibit good dispersion of Pt NPs (Figure S13, Supporting Information). ICP‐MS confirmed that the loading amounts of Pt cubes and Pt icosahedra on carbon carriers were 18.9% and 17.6%, respectively.

Due to meeting all the requirements for the determination of N_2_H_4_ intermediates, ECL signals of different electrocatalysts were collected after AOR. Typically, we focused on the ECL intensities of well‐known Pt‐based catalysts exposed to different crystal planes, with comparison of polycrystalline commercial Pt/C catalysts. ECL intensity of Pt(100)/C was high and altered significantly at different potentials, whereas that of Pt(111)/C was low and remained almost constant at all measured potentials (Figure 3e,f). Compared with commercial Pt/C catalysts (Figure 3g), Pt(100)/C exhibits higher ECL intensities, while Pt(111)/C has the lowest strength, suggesting the significant impact of structure sensitivity of Pt facets on the N_2_H_4_ intermediates during the electrocatalytic AOR. It is noteworthy that both Pt(100)/C and commercial Pt/C show the maximum ECL intensity at −0.16 V (vs Ag/AgCl), inferring that this potential is more advantageous to the formation of N_2_H_4_ intermediates (Figure 3h). Figure 3i depicts the CV curves of commercial Pt/C, Pt(100)/C, and Pt(111)/C catalysts, showing the obvious contrast of AOR current. Apparently, Pt(100)/C catalyst exhibits the maximum oxidation current at −0.13 V, while the Pt(111)/C catalyst shows a very weak oxidation current, further indicating that the performance of Pt(100)/C is superior to commercial Pt/C and Pt(111)/C. Combining the ECL intensity differences of three catalysts at different potentials, it is easy to infer that the formation of N_2_H_4_ intermediates explains why the electrocatalytic performance of Pt(100) crystal surface is more prominent than that of Pt(111) in AOR.

**Figure 4:**
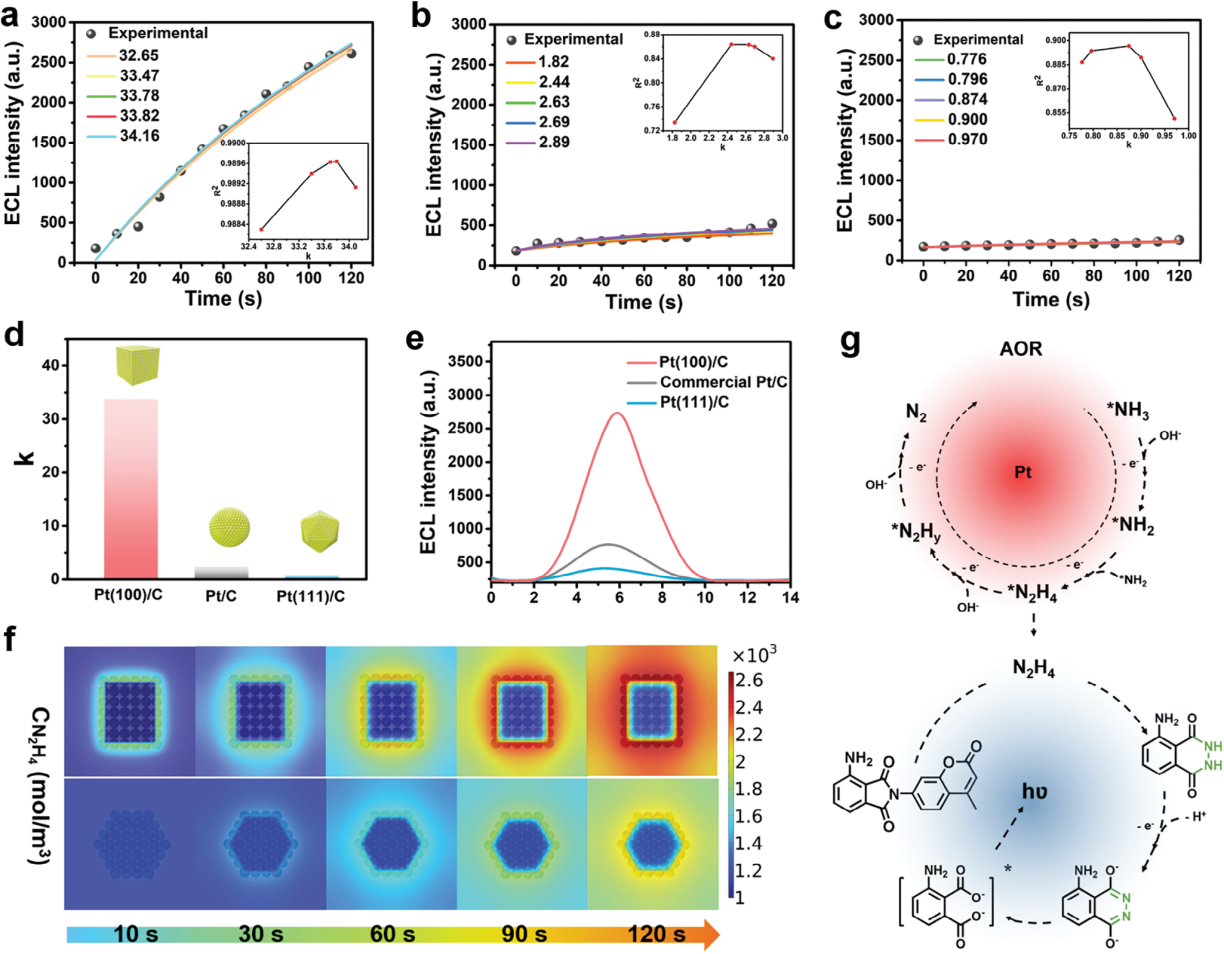
Time‐dependent experimental ECL data and simulated ECL curves (solid lines) of a) Pt(100)/C, b) commercial Pt/C and c) Pt(111)/C catalysts during AOR process. Inset: the coefficient of determination (R^2^) for experimental to simulated curves. d) Differences in the *k*‐values of the simulated functions of three types of Pt‐based catalysts exposed different crystal planes. e) Comparison of ECL signals of Pt(100)/C, Pt(111)/C and commercial Pt/C catalysts after 5 min of AOR process. f) Finite element simulation snapshots of N_2_H_4_ concentrations at different electrolysis time on Pt(100) and Pt(111) crystal planes. g) Mechanistic illustration of the N_2_H_4_ intermediate detection with ECL active probe CF in Pt catalyzed electrochemical AOR.

In addition to the quantitative determination of N_2_H_4_ intermediates of three electrocatalysts exposed different crystal planes at different potentials, the determination of N_2_H_4_ generation kinetics is also necessary for the overall assessment of performance of electrocatalyst and in‐depth comprehension of the complicated mechanism of AOR. For this purpose, time‐dependent ECL was supposed to provide insightful information. It should be noted that in order to more strictly compare the kinetic differences of Pt(100)/C, Pt(111)/C and commercial Pt/C catalysts. AOR was conducted at the same potential of −0.16 V (vs Ag/AgCl). **Figure** **4**a–c shows the ECL intensity as a function of time for Pt(100)/C, commercial Pt/C and Pt(111)/C electrocatalysts, respectively. Apparently, Pt(100)/C electrocatalysts exhibited stronger ECL intensities and faster ECL signal changes than the Pt(111)/C catalyst, implying the faster N_2_H_4_ generation kinetics of Pt(100)/C electrocatalysts. To more intuitively evaluate the crystal plane effect of the N_2_H_4_ intermediates generation kinetics of Pt‐based catalysts in electrocatalytic AOR, a series of simulated ECL curves were generated through function fitting. Equation 1 can effectively simulate the functional relationship of ECL intensity of three catalysts over time during AOR:

(1) $$I_{ECL}=a\times \ln\left(kt+b\right)+c$$

where *I_ECL_* is the overall ECL intensity, *t* is time, *k* represents the rate of change in ECL intensity over time, and a, b, c are the associated constants. a represents the weight of the linear relationship after logarithmic transformation, b and c represents the bias term of the linear relationship and nonlinear transformation, respectively. Equations (2), (3), (4) present the optimum fitting functional relationship of three Pt‐based catalysts with different exposed crystal planes:

(2) $$I_{Pt\left(100\right)}=3628.78\times \ln\left(33.47t+3702.18\right)-29780.25$$

(3) $$I_{Pt\left(111\right)}=99.58\times \ln\left(0.87t+106.55\right)-295.47$$

(4) $$I_{Pt/C}=159.14\times \ln\left(2.44t+78.18\right)-505.53$$

Significantly, the reaction between CF probe and N_2_H_4_ intermediate to generate luminol (LH) is the fundamental reason for the change of ECL intensity. The ECL mechanism of quantitative determination of N_2_H_4_ intermediate is outlined in Equations (5), (6), (7):

(5) $$CF+N_{2}H_{4}\to LH^{-}\left(in\;alkaline\;solution\right)+AMC$$

(6) $$LH^{-}+OH^{-}-e^{-}\to L^{\cdot -}+H_{2}O$$

(7) $$L^{\cdot -}+ROS\to Ap^{*}\to Ap+h\upsilon$$

Due to the constant concentration of reactive oxygen species (ROS) in the experiment, the amount of photon‐emitting species (Ap^*^) was positively correlated with that of LH. Moreover, CF is excessive throughout the entire measurement, so the amount of Ap^*^ is positively correlated with the concentration of N_2_H_4_ intermediate. In short, ECL intensity is positively correlated with the amount of N_2_H_4_ intermediate. Additionally, after CF reacted with N_2_H_4_ preconfigured with different concentrations, the ECL intensity showed a good linear positive correlation with N_2_H_4_ concentration (Figure 1c). Consequently, the *k* value not only represents the rate of change in ECL intensity over time, but also further explains the rate of change in the amount of N_2_H_4_ intermediates over time, which is an indispensable value for evaluating the kinetics of N_2_H_4_ intermediate formation. The higher the *k* value, the faster the generation rate of N_2_H_4_ intermediate and the faster the electrocatalytic AOR kinetics. For example, the optimal fit value for Pt(100)/C was determined from the highest coefficient of determination (R^2^, Figure 4a inset), corresponding to value of 33.82 (Figure 4a), while the best fit value for commercial Pt/C catalyst and Pt(111)/C was 2.44 and 0.87, respectively (Figure 4b,c). There is an obvious contrast in the *k*‐values of the simulated functions of Pt(100)/C, Pt(111)/C and commercial Pt/C electrocatalyst (Figure 4d). Specifically, Pt(100)/C has a much higher *k* value than Pt(111)/C and commercial Pt/C, further indicating that Pt(100) has superior kinetics for the generation of N_2_H_4_ intermediates. Figure 4e presents the comparison of ECL signals of Pt(100)/C, Pt(111)/C and commercial Pt/C catalysts after 5 min of AOR process, indicating the highest intensity of Pt(100) crystal planes. These experimental results consistently illustrate that the Pt(100) crystal planes exhibit superior performance in the generation of N_2_H_4_ intermediates than Pt(111) crystal planes, both in terms of the amount and the generation rate of N_2_H_4_.

Visualized 2D concentration profiles of N_2_H_4_ are showed in Figure 4f to better understand the Pt facets structural sensitivity of N_2_H_4_ intermediates during AOR process utilizing FES method. At different AOR times, the simulated N_2_H_4_ concentration on Pt(100) crystal surface is much higher than that on the Pt(111) surface. Especially, this concentration difference is already very obvious when AOR reaches 120 s and consistent with the ECL measurement results. These results well support the high generation efficiency of N_2_H_4_ intermediates on the Pt(100) crystal surface, further explaining why the electrocatalytic performance of Pt(100) crystal surface is more prominent than that of Pt(111) in AOR.

Based on the above experimental and theoretical results, a possible mechanism for the detection of N_2_H_4_ intermediates during AOR using ECL signals was proposed (Figure 4g). First, AOR happened under the catalysis of Pt and produced N_2_H_4_ intermediate, which rapidly reacted with CF to generate luminol. Then, the luminol anions generated by luminol under alkaline conditions were further electrochemically oxidized to luminol anion radicals during the anodic potential scanning, and the H_2_O_2_ in the system was catalyzed to generate a large amount of ROS; next, ROS reacted with luminol anion through radicals to form an excited‐state intermediate 3‐aminophthalate anion, resulting in significant ECL emission.

On the basis of the previous findings, the probe is capable of generating AMC and luminol following a Gabriel type‐based hydrazinolysis in the presence of N_2_H_4_.^[^ ^43^ ^]^ Luminol has been cleverly used here as an ECL signal molecule to observe the formation of N_2_H_4_ during AOR. Naturally, due to the high quantum yield of AMC,^[^ ^43^ ^]^ fluorescence monitoring was further performed to verify the N_2_H_4_ produced in the AOR process. Figure S14 (Supporting Information) shows the UV–vis spectra of CF in solution of H_2_O/DMSO (3:7, v/v). Fluorescence monitoring was further utilized to validate the generatation of N_2_H_4_ intermediate during AOR process. During the electrocatalytic AOR, a noticeable reduction in the intensity of emission at 480 nm was detected when compared to the initial concentration of the CF solution. In addition, a novel emission emerged at the wavelength of 430 nm. This result is consistent with fluorescence change caused by the addition of N_2_H_4_, further confirming the generation of N_2_H_4_ throughout the electrocatalytic AOR (Figures S15 and S16, Supporting Information). The CIE coordinate diagram distinctly illustrated the change of luminescence (Figure S17, Supporting Information). In a word, the fluorescence study of the N_2_H_4_ intermediates provides cogent experimental evidence for deeply understanding of electrchemical AOR mechanism.

We next investigated the reaction product between CF and N_2_H_4_ by using mass and HPLC spectra. As shown in Figure S18 (Supporting Information), the reaction product of CF and N_2_H_4_ is represented by the mass spectra of fragments of m/z = 176.10 and 178.09, which is attributable to the fragments [AMC + H]^+^ and [luminol + H]^+^, respectively. In addition, after 20 min of AOR process at −0.16 V (vs Ag/AgCl) of three Pt‐based catalysts with different crystal planes, the mass spectrum of the reaction between AOR electrolyte (0.1 m KOH + 0.1 m NH_3_) and CF was further tested. The fragment of m/z = 178.9 is attributable to the fragments of [luminol + H]^+^ (Figure S19, Supporting Information). HPLC analysis further certified the reaction products of AMC and luminol (Figure S20, Supporting Information). The above results further indicated that AMC and luminol were formed during AOR process in the presence of the probe CF.

**Figure 5:**
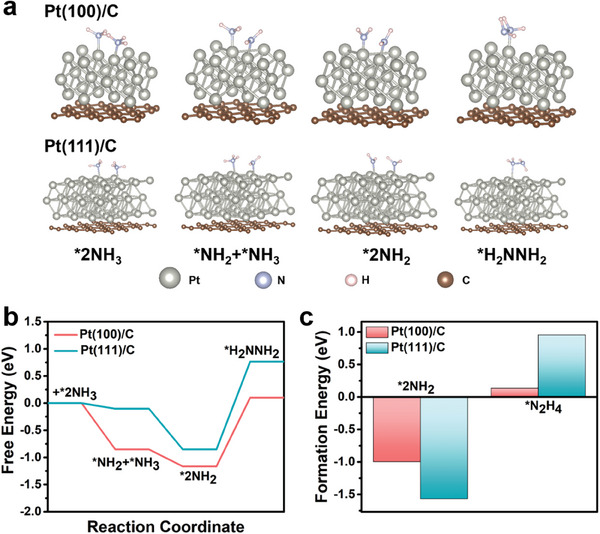
a) Transition state images of ^*^NH_3_ to ^*^N_2_H_4_ on Pt(100)/C and Pt(111)/C. b) Free energy diagram of electrochemical ammonia oxidation on Pt/C with different exposed crystals. c) Formation energies (eV) of adsorbates.

To further verify the structural sensitivity of Pt crystal planes in the generation kinetics of the N_2_H_4_ intermediates during AOR, DFT calculated Gibbs free energies of AOR steps at Pt(100)/C and Pt(111)/C interfaces. The calculated structure model (**Figure** **5**a) and Gibbs free energy distribution (Figure 5b) of N_2_H_4_ produced in AOR process on Pt(100)/C and Pt(111)/C were further presented. Comparing the two surfaces, Pt(100)/C represents the stronger binding affinity for ^*^NH_2_ and the larger release energy of ^*^NH_3_ to ^*^NH_2_, indicating that the generation rate of ^*^NH_2_ on Pt(100)/C may be faster. In addition to stronger binding with ^*^NH_2_, Pt(100)/C also has stronger binding with ^*^N_2_H_4_. Figure 5c reveals the formation energy of N_2_H_4_ on Pt(100)/C and Pt(111)/C during AOR. ^*^NH_2_ is a candidate for participation in the proposed rate‐determining step involving dimerization to form N─N bonds. The formation energy of ^*^NH_2_ and ^*^N_2_H_4_ on Pt(100)/C is significantly lower than that on Pt(111)/C, that is, Pt(100)/C has faster N_2_H_4_ formation rate than Pt(111)/C, resulting in a significant difference in AOR reaction kinetics. Obviously, the theoretical calculation is consistent with the ECL analysis results, which further indicates that the ECL system is reliable and capable of quantitative detection of N_2_H_4_ intermediate and further determined the formation kinetics of N_2_H_4_ intermediate in the AOR process.

Combined with ECL analysis, FL spectrum and CV data, N_2_H_4_ is clearly demonstrated to be an electrochemical AOR intermediates. As a consequence of these findings, Pt‐based electrocatalysts in alkaline media exhibit the following AOR mechanism under the framework of Gerischer and Mauerer mechanism:

(8) $$NH_{3\left(aq\right)}\to NH_{3\left(ads\right)}$$

(9) $$NH_{3\left(ads\right)}↔NH_{2\left(ads\right)}+H^{+}+e^{-}$$

(10) $$NH_{2\left(ads\right)}+NH_{2\left(ads\right)}\to N_{2}H_{4\left(ads\right)}$$

(11) $$N_{2}H_{4\left(ads\right)}+4OH^{-}\to N_{2}+4H_{2}O+e^{-}$$

(12) $$NH_{2\left(ads\right)}↔NH_{\left(ads\right)}+H^{+}+e^{-}$$

(13) $$NH_{\left(ads\right)}\to N_{\left(ads\right)}+H^{+}+e^{-}$$

Initially, the adsorption of ammonia occurs on the catalyst surface (Equation 8), followed by continuous dehydrogenation (Equation 9). Then, with further dimerization occurring, N_2_H_4_, an active intermediate during AOR, is formed by NH_2(ads)_ coupling in Equation 10. Next, N_2_H_4_ is continuously dehydrogenated to form the final product of AOR, N_2_, which is the principal component of the AOR peak (Equation 11). Equations 12 and 13 are supposed to represent side reactions that lead to the production of the poison N_ads_, which is responsible for deactivating the electrocatalyst.^[^ ^50^, ^51^ ^]^ More importantly, ECL analysis, DFT calculation and FES snapshots all indicate that N_2_H_4_ formation rate is faster on Pt(100) than Pt(111) interface. Research to date has identified the following factors as being responsible for the more outstanding AOR performance of Pt(100) crystal face: i) The crystal face of Pt(100) is more conducive to the formation of ^*^NH_2_, ii) The dimerization reaction energy between ^*^NH_2_ is lower on the Pt(100) surface; iii) the electrochemical reaction may be accelerated by the adsorption of OH, and the ^*^OH is thermodynamically stable on Pt(100) surface.^[^ ^8^ ^]^

## 3 Conclusion

In summary, this study focuses on the synthesis of a ECL active probe (CF) for the in‐depth study of the formation kinetics of the N_2_H_4_ intermediates on Pt catalysts with different crystal planes during AOR by using time‐resolved ECL technology. The obtained CF probe could fast and selectively react with N_2_H_4_, releasing luminol that is used as the ECL probe. On this basis, the probe is capable of reporting the level of N_2_H_4_ in real time during AOR by ECL. Time‐resolved ECL analysis indicates that Pt(100) facet is found to exhibit superior N_2_H_4_ formation kinetics compared to Pt(111) facet, which is consistent with DFT calculations results. The FES presented a higher two‐dimensional concentration distribution on Pt(100) facet than Pt(111) facet, further verifying the faster N_2_H_4_ formation rate on the Pt(100) facet. Time‐dependent fluorescence spectrum further confirmed the dynamic changes of the generated N_2_H_4_ intermediate. All these results reveal the fundamental reason for the superior reaction kinetics of the Pt(100) surface over the Pt(111) surface during AOR. Accordingly, a mechanism by which N_2_H_4_ as an active intermediate lead to the main product N_2_ was proposed under the framework of Gerischer–Mauerer. Therefore, this ECL active molecular probe offers not only a promising detection tool for evaluating formation kinetics of the N_2_H_4_ intermediate on catalysts with different crystal faces in electrochemistry but also opens new doors for in‐depth understanding of AOR mechanisms and the design of efficient electrocatalysts in energy conversion and storage applications.

## 4 Experimental Section

### Electrochemical and ECL Measurement

All electrochemical measurements were performed using a three‐electrode system controlled by the CHI 760E electrochemical analyzer system or ECL MPI‐E II electrochemical and chemiluminescence analysis system ECL. In all experiments, Ag/AgCl and Pt wires were used as reference and counter electrodes, respectively. A reserve solution of 2 mm CF was prepared in water and DMSO (v/v = 3:7). The homogeneous dispersion of Pt/C catalyst with different crystallographic planes was prepared by mixing 2 mg mL^−1^ Pt/C catalyst dispersed in mixed solvent of isopropanol and DI water with 5% Nafion solution. Cyclic voltammetry (CV) was performed in the potential window of −1 to 0.5 V (vs Ag/AgCl) at 50 mV s^−1^ scan rate. For quantitative detection of N_2_H_4_ intermediates during electrochemical AOR through ECL signals, electrolytic reaction solution (50 µL) at different times were extracted and reacted with 10 µL of CF probe (2 mm) immediately, then ECL process were carried out in the electrolytic cell (0.01 m PBS + 0.05 mm H_2_O_2_). The potential range of ECL was from 0 to 0.8 V (vs Ag/AgCl) at 50 mV s^−1^ scan rate, and the photomultiplier tube (PMT) was set at 800 V.

### Synthesis of Pt Cubes

40 mg Pt(acac)_2_ were dissolved in 5 mL benzyl ether, 3.68 mL oleylamine and 0.65 mL oleic acid under N_2_ atmosphere. The reaction flask was then placed into a preheated oil bath. A solution of 4 mg Mn_2_(CO)_10_ in 0.5 mL chloroform was injected into the reaction mixture at 160 °C, and the reaction was allowed to heated to 240 °C. After 30 min of reaction at 240 °C, the solution was cooled down and the products were isolated by adding ethanol and centrifugation. The NCs were washed with hexane three times and redispersed in hexane.

### Synthesis of Pt Icosahedron

40 mg Pt(acac)_2_ were dissolved in 5 mL benzyl ether, 3.68 mL oleylamine and 0.65 mL oleic acid under N_2_ atmosphere. The reaction flask was then placed into a preheated oil bath. A solution of 4 mg Mn_2_(CO)_10_ in 0.5 mL chloroform was injected into the reaction mixture at 160 °C, and the reaction was allowed to heated to 190 °C. After 30 min of reaction at 190 °C, the solution was cooled down and the products were isolated by adding ethanol and centrifugation. The NCs were washed with hexane (containing 1% oleylamine) three times and redispersed in hexane.

### Synthesis of Pt(100)/C and Pt(111)/C Catalyst

Carbon blank (Vulcan XC‐72) was used as the support for Pt(100)/C and Pt(111)/C catalyst. For the preparation of Pt/C catalyst materials, the amount of carbon and Pt was calculated to obtain 35 mg of carbon‐supported 20 wt.% Pt/C catalyst. In a standard procedure, 28 mg of carbon blank was soaked 24 h and dispersed by ultrasound for 1 h. Then, 7 mg Pt icosahedral or cubes in 3 mL hexyl hydride were added and stirred 24 h. The as‐prepared catalyst materials were centrifuged and washed three times with acetone and deionized water, followed by vacuum drying at 120 °C for 12 h to obtain the final product.

### Synthesis of CF (4‐phtalamide‐N‐(4′‐methylcoumarin) naphthalimide)

Before CF synthesis, compound 1 was synthesized. 2.59 mmol 3‐nitrophthalic anhydride (500 mg) and 2.59 mmol 7‐amino‐4‐methylcoumarin (AMC) (454.1 mg) were added to a round‐bottom flask containing 50 mL acetic acid and heated to reflux for 4 h. After the reaction was cooled to room temperature, the precipitate was filtered and washed three times with ethanol. Yellow solid product 1 does not need further purification and was directly used in the next reaction. 1 (0.5 g, 1.42 mmol), Pd/C (5%, 0.05 g), and methanol with 5% DMSO (30 mL) were added to a thick‐walled pressure flask with a guiding air interface and then sealed. The reaction was fed into H_2_ (1.0 MPa) and stirred overnight at room temperature. The resulting yellow solid powder CF was filtered and washed three times with water.

## Conflict of Interest

The authors declare no conflict of interest.

## Supporting information

Supporting Information

## Data Availability

The data that support the findings of this study are available from the corresponding author upon reasonable request.

## References

[1] W. Guo , K. Zhang , Z. Liang , R. Zou , Q. Xu , Chem. Soc. Rev. 2019, 48, 5658. 31742279 10.1039/c9cs00159j

[2] H. Park , K.‐H. Choo , H.‐S. Park , J. Choi , M. R. Hoffmann , Chem. Eng. J. 2013, 215–216, 802.

[3] M. Zhang , P. Zou , G. Jeerh , S. Chen , J. Shields , H. Wang , S. Tao , ACS Sustainable Chem. Eng. 2020, 8, 12817.

[4] X. Zhang , F. Zhu , L. Chen , Q. Zhao , G. Tao , Bioresour. Technol. 2013, 146, 161. 23933023 10.1016/j.biortech.2013.07.024

[5] K. Siddharth , Y. Hong , X. Qin , H. J. Lee , Y. T. Chan , S. Zhu , G. Chen , S.‐I. Choi , M. Shao , Appl. Catal. B Environ. 2020, 269, 118821.

[6] S. Gottesfeld , J. Electrochem. Soc. 2018, 165, J3405.

[7] N. J. Bunce , D. Bejan , Electrochim. Acta 2011, 56, 8085.

[8] X. Xi , Y. Fan , K. Zhang , Y. Liu , F. Nie , H. Guan , J. Wu , Chem. Eng. J. 2022, 435, 134818.

[9] L. A. Diaz , G. G. Botte , Electrochim. Acta 2015, 179, 519.

[10] D. Skachkov , C. Venkateswara Rao , Y. Ishikawa , J. Phys. Chem. C 2013, 117, 25451.

[11] F. J. Vidal‐Iglesias , J. Solla‐Gullón , J. M. Pérez , A. Aldaz , Electrochem. Commun. 2006, 8, 102.

[12] H. J. J. E. C. Gerischer , J. Electroanal. Chem. 1970, 25, 421.

[13] S. W. Wallace , I. T. McCrum , M. J. Janik , Catal. Today 2021, 371, 50.

[14] K. Yang , J. Liu , B. Yang , ACS Catal. 2021, 11, 4310.

[15] M. Duca , M. O. Cucarella , P. Rodriguez , M. T. M. Koper , J. Am. Chem. Soc. 2010, 132, 18042. 21141817 10.1021/ja1092503

[16] X. Lin , X. Zhang , Z. Wang , X. Zhu , J. Zhu , P. Chen , T. Lyu , C. Li , Z. Qun Tian , P. Kang Shen , J. Colloid Interface Sci. 2021, 601, 1. 34052723 10.1016/j.jcis.2021.04.068

[17] E. Bertin , C. Roy , S. Garbarino , D. Guay , J. Solla‐Gullón , F. J. Vidal‐Iglesias , J. M. Feliu , Electrochem. Commun. 2012, 22, 197.

[18] Y. Katayama , T. Okanishi , H. Muroyama , T. Matsui , K. Eguchi , ACS Catal. 2016, 6, 2026.

[19] F. Shi , P. N. Ross , H. Zhao , G. Liu , G. A. Somorjai , K. Komvopoulos , J. Am. Chem. Soc. 2015, 137, 3181. 25689135 10.1021/ja5128456

[20] T. Matsui , S. Suzuki , Y. Katayama , K. Yamauchi , T. Okanishi , H. Muroyama , K. Eguchi , Langmuir 2015, 31, 11717. 26447852 10.1021/acs.langmuir.5b02330

[21] H. Zhang , X.‐G. Zhang , J. Wei , C. Wang , S. Chen , H.‐L. Sun , Y.‐H. Wang , B.‐H. Chen , Z.‐L. Yang , D.‐Y. Wu , J.‐F. Li , Z.‐Q. Tian , J. Am. Chem. Soc. 2017, 139, 10339. 28700232 10.1021/jacs.7b04011

[22] X.‐M. Lin , X.‐T. Wang , Y.‐L. Deng , X. Chen , H.‐N. Chen , P. M. Radjenovic , X.‐G. Zhang , Y.‐H. Wang , J.‐C. Dong , Z.‐Q. Tian , J.‐F. Li , Nano Lett. 2022, 22, 5544. 35699945 10.1021/acs.nanolett.2c01744

[23] D.‐Y. Wei , M.‐F. Yue , S.‐N. Qin , S. Zhang , Y.‐F. Wu , G.‐Y. Xu , H. Zhang , Z.‐Q. Tian , J.‐F. Li , J. Am. Chem. Soc. 2021, 143, 15635. 34541841 10.1021/jacs.1c04590

[24] J. Liu , K. Yu , H. Zhang , J. He , J. Jiang , H. Luo , Chem. Sci. 2021, 12, 9494. 34349924 10.1039/d1sc01385h PMC8278903

[25] B. Michalak , B. B. Berkes , H. Sommer , T. Bergfeldt , T. Brezesinski , J. Janek , Anal. Chem. 2016, 88, 2877. 26813026 10.1021/acs.analchem.5b04696

[26] T. Li , S. Han , C. Wang , Y. Huang , Y. Wang , Y. Yu , B. Zhang , ACS Catal. 2021, 11, 14032.

[27] K. Zhu , X. Zhu , W. Yang , Angew. Chem., Int. Ed. 2018, 58, 1252. 10.1002/anie.201802923 29665168

[28] A. M. Chaparro , Electrochim. Acta 2021, 372, 137856.

[29] Z.‐Q. Wu , J.‐J. Liu , J.‐Y. Li , D. Xu , X.‐H. Xia , Anal. Chem. 2017, 89, 12924. 29110460 10.1021/acs.analchem.7b03780

[30] G. Chen , H. Liu , Environ. Sci. Technol. 2017, 51, 11643. 28902987 10.1021/acs.est.7b02021

[31] C. Zong , C. Zhang , P. Lin , J. Yin , Y. Bai , H. Lin , B. Ren , J.‐X. Cheng , Chem. Sci. 2021, 12, 1930. 10.1039/d0sc05132b PMC8179047 34163957

[32] Y. Song , S. Lu , J. Hai , K. Liang , S. Sun , G. Meng , B. Wang , Anal. Chem. 2021, 93, 11470. 34379390 10.1021/acs.analchem.1c01497

[33] H. Peng , Z. Huang , H. Deng , W. Wu , K. Huang , Z. Li , W. Chen , J. Liu , Angew. Chem., Int. Ed. 2020, 59, 9982. 10.1002/anie.201913445 31691480

[34] H. Xia , X. Zheng , J. Li , L. Wang , Y. Xue , C. Peng , Y. Han , Y. Wang , S. Guo , J. Wang , E. Wang , J. Am. Chem. Soc. 2022, 144, 7741. 35438986 10.1021/jacs.2c00865

[35] M. Saqib , S. Bashir , H. Li , C. Li , S. Wang , Y. Jin , Anal. Chem. 2019, 91, 12517. 31502437 10.1021/acs.analchem.9b03314

[36] M. Yang , Y. Kostov , H. A. Bruck , A. Rasooly , Anal. Chem. 2008, 80, 8532. 18855418 10.1021/ac801418n PMC2845180

[37] Y. He , J. Du , J. Luo , S. Chen , R. Yuan , Biosens. Bioelectron. 2020, 150, 111898. 31767347 10.1016/j.bios.2019.111898

[38] A. Zanut , F. Palomba , M. Rossi Scota , S. Rebeccani , M. Marcaccio , D. Genovese , E. Rampazzo , G. Valenti , F. Paolucci , L. Prodi , Angew. Chem., Int. Ed. 2020, 59, 21858. 10.1002/anie.202009544 33000888

[39] W. Wei , H. Lin , T. Hao , X. Su , X. Jiang , S. Wang , Y. Hu , Z. Guo , Sens. Actuators, B 2021, 332, 129525.

[40] Q. Wang , M. Goetsch , J. Crohns Colitis 2019, 13, S096.

[41] S. Y. Xiao , S. J. Zhen , C. Z. Huang , Y. F. Li , Biosens. Bioelectron. 2021, 186, 113263. 33964795 10.1016/j.bios.2021.113263

[42] M.‐X. Li , Q.‐M. Feng , Z. Zhou , W. Zhao , J.‐J. Xu , H.‐Y. Chen , Anal. Chem. 2018, 90, 1340. 29250961 10.1021/acs.analchem.7b04307

[43] L. Cui , C. Ji , Z. Peng , L. Zhong , C. Zhou , L. Yan , S. Qu , S. Zhang , C. Huang , X. Qian , Y. Xu , Anal. Chem. 2014, 86, 4611. 24702027 10.1021/ac5007552

[44] K. Siddharth , P. Alam , M. D. Hossain , N. Xie , G. S. Nambafu , F. Rehman , J. W. Y. Lam , G. Chen , J. Cheng , Z. Luo , G. Chen , B. Z. Tang , M. Shao , J. Am. Chem. Soc. 2021, 143, 2433. 33507070 10.1021/jacs.0c13178

[45] X.‐L. Huo , N. Zhang , H. Yang , J.‐J. Xu , H.‐Y. Chen , Anal. Chem. 2018, 90, 13723. 30354080 10.1021/acs.analchem.8b04141

[46] Y. Hao , Y. Li , J. Wu , L. Meng , J. Wang , C. Jia , T. Liu , X. Yang , Z.‐P. Liu , M. Gong , J. Am. Chem. Soc. 2021, 143, 1493. 33439638 10.1021/jacs.0c11307

[47] Y. Li , X. Li , H. S. Pillai , J. Lattimer , N. Mohd Adli , S. Karakalos , M. Chen , L. Guo , H. Xu , J. Yang , D. Su , H. Xin , G. Wu , ACS Catal. 2020, 10, 3945.

[48] Y. Kang , J. B. Pyo , X. Ye , R. E. Diaz , T. R. Gordon , E. A. Stach , C. B. Murray , ACS Nano 2013, 7, 645. 23211025 10.1021/nn3048439

[49] J. R. Barbosa , M. N. Leon , C. M. Fernandes , R. M. Antoniassi , O. C. Alves , E. A. Ponzio , J. C. M. Silva , Appl. Catal. B Environ. 2020, 264, 118458.

[50] J. F. E. Gootzen , A. H. Wonders , W. Visscher , R. A. van Santen , J. A. R. van Veen , Electrochim. Acta 1998, 43, 1851.

[51] A. C. A. de Vooys , M. T. M. Koper , R. A. van Santen , J. A. R. van Veen , J. Electroanal. Chem. 2001, 506, 127.

