# Supplementary material for: Discovering Facet‐Dependent Formation Kinetics of Key Intermediates in Electrochemical Ammonia Oxidation by a Electrochemiluminescence Active Probe
Source: Adv Sci (Weinh). 2024 Jun 23;11(32):2402673. doi: 10.1002/advs.202402673 (PMC11348187; doi:10.1002/advs.202402673)
Supplement: Supplementary file 1 — Supporting Information [file ADVS-11-2402673-s001.pdf]

ADVANCED SCIENCE
Open Access

Supporting Information

for *Adv. Sci.*, DOI 10.1002/advs.202402673

Discovering Facet-Dependent Formation Kinetics of Key Intermediates in Electrochemical Ammonia Oxidation by a Electrochemiluminescence Active Probe

Dina Sun, Jiaqi Zhang, Heng Wang, Yanxia Song, Jing Du, Genping Meng, Shihao Sun, Weihua Deng, Zhiyi Wang and Baodui Wang**

Supporting Information

DOI: 10.1002/((please add manuscript number))

Article type: Article

Discovering Facet-Dependent Formation Kinetics of Key Intermediates in Electrochemical Ammonia Oxidation by a Electrochemiluminescence Active Probe

Dina Sun,[a] Jiaqi Zhang,[a] Heng Wang,[c] Yanxia Song,[a] Jing Du,[a] Genping Meng[a], Shihao Sun,[a] Weihua Deng,[c] Zhiyi Wang,[b] and Baodui Wang*[a]*

D. Sun, J. Zhang, Y. Song, J. Du, G. Meng, S. Sun, Prof. B. Wang.

State Key Laboratory of Applied Organic Chemistry, Key Laboratory of Nonferrous Metal Chemistry and Resources Utilization of Gansu Province, Lanzhou University, Lanzhou, Gansu 730000, China.

E-mail: wangbd@lzu.edu.cn

Prof. Z. Wang

Spin-X Institute, School of Chemistry and Chemical Engineering, State Key Laboratory of Luminescent Materials and Devices, South China University of Technology, Guangzhou 511442, China.

E-mail: wangzhiyi2020@scut.edu.cn

H. Wang, Prof. W. Deng

School of Mathematics and Statistics, Gansu Key Laboratory of Applied Mathematics and Complex Systems, Lanzhou University, Lanzhou 730000, China.

Experimental Section

Materials and Instrument. The chemicals and solvents were obtained from commercial sources and were used directly without further purification unless specified. 7-Amino-4-methylcoumarin (AMC), 3-Nitropropionic phthalic anhydride, commercial Pt/C (20%) catalysts were procured from Shanghai Macklin Biochemical Co., Ltd. Ammonium hydroxide, $Pt(acac)_2$ (97%), benzyl ether (98%), oleylamin (70%), oleic acid and $Mn_2(CO)_{10}$ were purchased from Sigma-Aldrich, hydrazine hydrate, potassium hydroxide, acetic acid, ethanol, ethyl acetate, and hexane were purchased from Lianlong Tianjin Pharmaceutical Chemical Co. Ltd. Hydrogen peroxide (30%) and isopropanol were provided by Aladdin reagents.

CF was synthesized and purified by column chromatography and characterized by ^1H NMR. ^1H NMR spectrum was recorded on a Bruker AV 400 Spectrometer at 400 MHz in DMSO-*d6*. Tetramethylsilane was used as the internal standard.MS spectra was recorded on a micro TOF II instrument. UV-vis absorbance measurements were recorded on Shimadzu UV-1750. Fluorescence spectra were recorded by a Shimadzu RF-5301 spectrofluorophotometer. Transmission electron microscopy (TEM) and high-resolution TEM investigations were carried out on a FEI Talos 200s electron microscope operating at an accelerating voltage of 200 kV. Powdered X-ray diffraction (PXRD) patterns were recorded on AXS D8-Advanced diffractometer with Cu Ka radiation. Time-dependent fluorescent images are recorded utilizing VISQUE Invivo Smart-LF (Vieworks, Korea). ECL measurements were carried out on an MPI-E II electrochemical and chemiluminescent analytical system (Xi'an Remex Analytical Instrument Co., Ltd, China). All electrochemical measurements were performed using a three-electrode system controlled by the CHI 760E (Chenhua, Shang Hai) electrochemical analyzer system.

Theoretical Calculations. We have employed the first-principles[1, 2] to perform all spin-polarization density functional theory (DFT) calculations within the generalized gradient approximation (GGA) using the Perdew-Burke-Ernzerhof (PBE)[3] formulation. The projected augmented wave (PAW) potentials[4, 5] were chosen to describe the ionic cores and take valence electrons into account using a plane wave basis set with a kinetic energy cutoff of 450 eV. Partial occupancies of the Kohn−Sham orbitals were allowed using the Gaussian smearing method and a width of 0.05 eV. The electronic energy was considered self-consistent when the energy change was smaller than 10^{-4} eV. A geometry optimization was considered convergent

when the energy change was smaller than 0.05 eV Å^{-1}. Finally, the adsorption energies (E_{ads}) were calculated as:

$$E_{ads} = E_{ad/sub} - E_{sub} \qquad (1)$$

where $E_{ad/sub}$, E_{ad}, and E_{sub} are the total energies of the optimized adsorbate/substrate system, the adsorbate in the gas phase, and the clean substrate, respectively. The Brillouin zone integral uses the surfaces structures of 3×3×1 monkhorst pack K-point sampling for structures. The free energies of elemental reaction steps were calculated by the computational hydrogen electrode model developed by Nørskov et al. The free energy (ΔG) for elemental reaction step were calculated as:

$$\Delta G = \Delta E + \Delta EZPE - T\Delta S \qquad (2)$$

where ΔE is the difference between the total energy, ΔEZPE and ΔS are the differences in the zero-point energy and the change of entropy, T is the temperature (T =300 K in this work), respectively. The U correction had been used for our systems.

Photoluminescence spectra. Fluorescence spectra were recorded by a Shimadzu RF-5301 spectrofluorophotometer. The excitation wavelength (λ_{ex}) was fixed at 380 nm and the range of emission wavelength was set between 390 to 650 nm for all the obtained photoluminescence (PL) spectral data. In order to monitor N_2H_4 intermediates produced in the electrochemical AOR process (Pt(100)/C as the catalyst), the electrochemical reaction solution (10 μL) at different reaction moment was added to CF solution (2 mL, 2 μM) and allowed to stand for 2 min to complete the reaction. The fluorescence spectra were then immediately recorded.

COMSOL Simulation Section

The modeling of the tandem N_2H_4 accumulation-diffusion process was performed in COMSOL Multiphysics software with diluted species transport module. Models of electrode geometries including planar and porous electrodes were built. The domain equation is the diffusion equation, known as Fick's 2nd law, which describes the chemical transport of the electroactive species：

$$\frac{\partial c_i}{\partial t} + \nabla \cdot J_i + \mathrm{u} \cdot \nabla c_i = R_i \qquad (3)$$

$$J_i = -D_i \cdot \nabla c_i \qquad (4)$$

At the bulk boundary, a uniform concentration was assumed to equal to the bulk concentration for the reactants, where the products have the initial concentration of 1000 mol/m^3. Under the action of catalyst, reactants are constantly produced at the catalyst boundary. The control equation of reactants is shown below:

$$NH_{3(aq)} \longrightarrow NH_{3(ads)}$$

$$NH_{3(ads)} \longleftrightarrow NH_{2(ads)} + H^+ + e^-$$

$$NH_{2(ads)} + NH_{2(ads)} \longrightarrow N_2H_{4(ads)}$$

The control equations for electrochemical reactions and concentration distribution are as follows. It can be seen that electrochemical reactions act as source terms, providing reactants to the diffusion equation. The reaction rate follows the Arrhenius reaction formula:

$$\frac{\partial c_i}{\partial t} + \nabla \cdot J_i + \mathrm{u} \cdot \nabla c_i = R_i + S_i \qquad (5)$$

$$k = Ae^{-E_a/RT} \qquad (6)$$

where c is the concentration, J represents the flux of diffusion, R is the reaction rate, k is the rate constant; A is the characteristic constant of the given reaction, known as the pre-exponential factor; e is the base of natural logarithm (2.718); R is the gas constant (8.314 J mol^{-1} K^{-1}), E_a is the activation energy of the reaction. Different reactants have different reaction rates under different catalysts. By applying different reaction rates, different reactant concentrations can be obtained. Specifically, Pt(100) crystal catalysts have higher reaction rates, thus possessing higher reactant flux terms, and the surrounding reactant concentrations are also higher.

Table S1. Simulation parameters

Name	Value	Description
D (N_2H_4)	3 e^{-11} m^2 s^{-1}	diffusion coefficient of N_2H_4
C (N_2H_4)	1000 mol m^{-3}	initial concentration of N_2H_4
k	150 mol m^{-3} s^{-1}	reaction rate

T	293 K	temperature
α	0.5	electron transport coefficient
R	8.314 J mol^{-1} k^{-1}	ideal gas constant
F	96485 C mol^{-1}	Faraday constant

Supplementary Figures

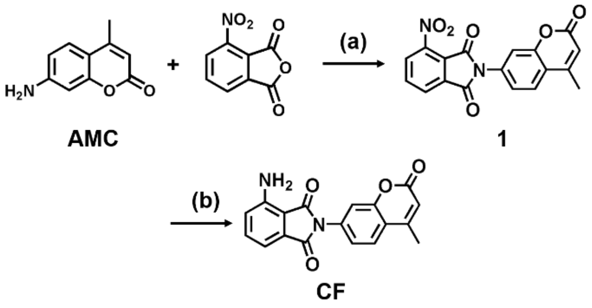

Figure S1 Schematic diagram for the formation of CF. (a) 3-Nitropropionic phthalic anhydride, AMC, acetic acid, reflex, overnight; (b) Compound 1, Pd/C, H_2 (1.0 MPa), room temperature, overnight.

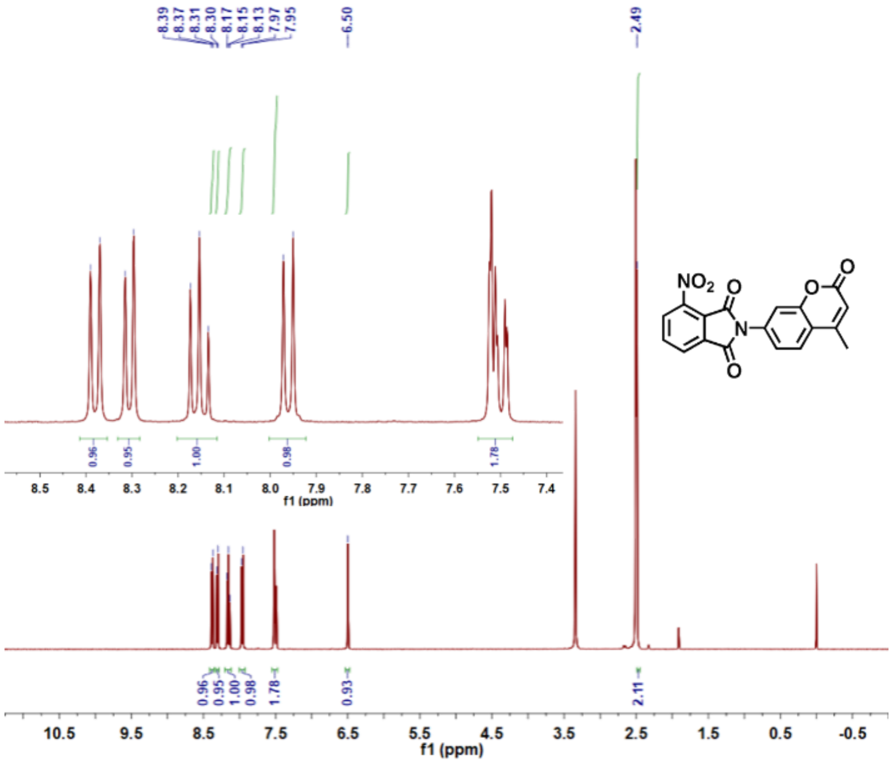

Figure S2 ^{1}H NMR spectra of compound 1 in DMSO-*d6*. ^{1}H NMR (400 MHz, DMSO-*d6*) ppm δ 8.38 (d, *J* = 8.1 Hz, 1H), 8.31 (d, *J* = 7.4 Hz, 1H), 8.15 (t, *J* = 7.8 Hz, 1H), 7.96 (d, *J* = 8.4 Hz, 1H), 7.55 – 7.47 (m, 2H), 6.50 (s, 1H), 2.49 (s, 2H).

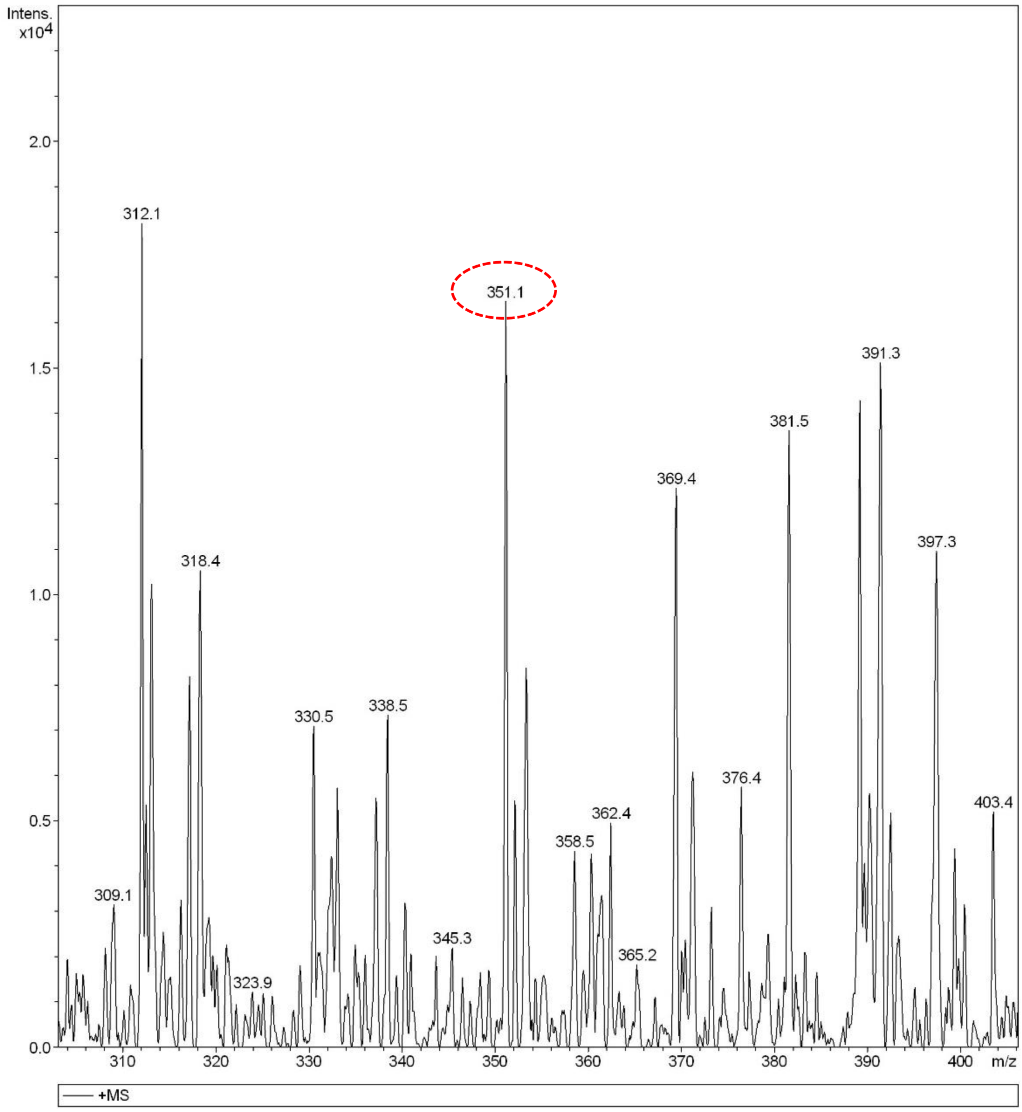

Figure S3 MS of the **1** molecule. Its molecular weight is 350.1.

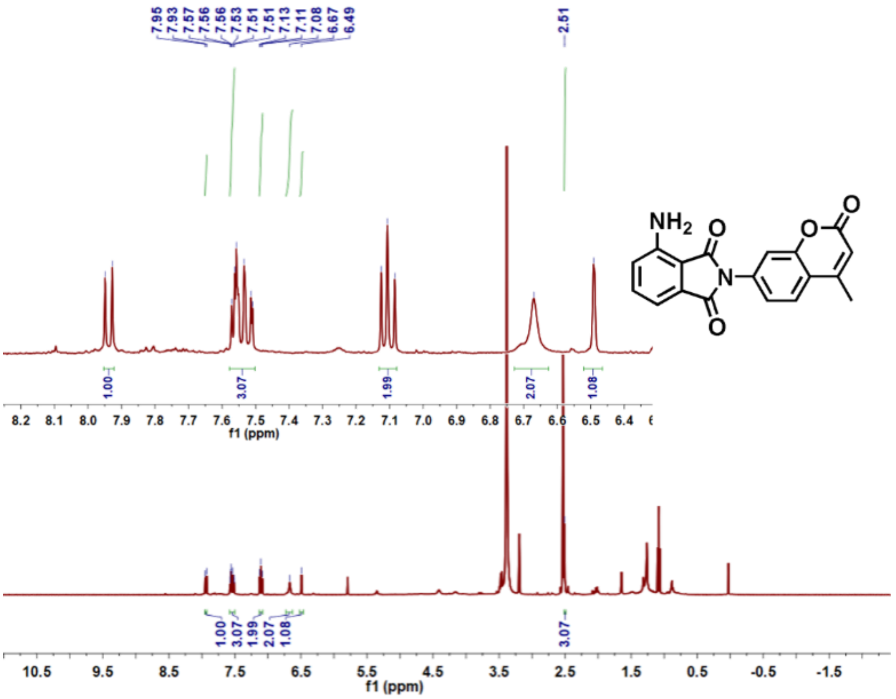

Figure S4 ^{1}H NMR spectra of **CF** in DMSO-*d6*. ^{1}H NMR (400 MHz, DMSO-*d6*) ppm δ 7.94 (d, *J* = 8.5 Hz, 1H), 7.58 – 7.50 (m, 3H), 7.11 (t, *J* = 8.2 Hz, 2H), 6.67 (s, 2H), 6.49 (s, 1H), 2.51 (s, 3H).

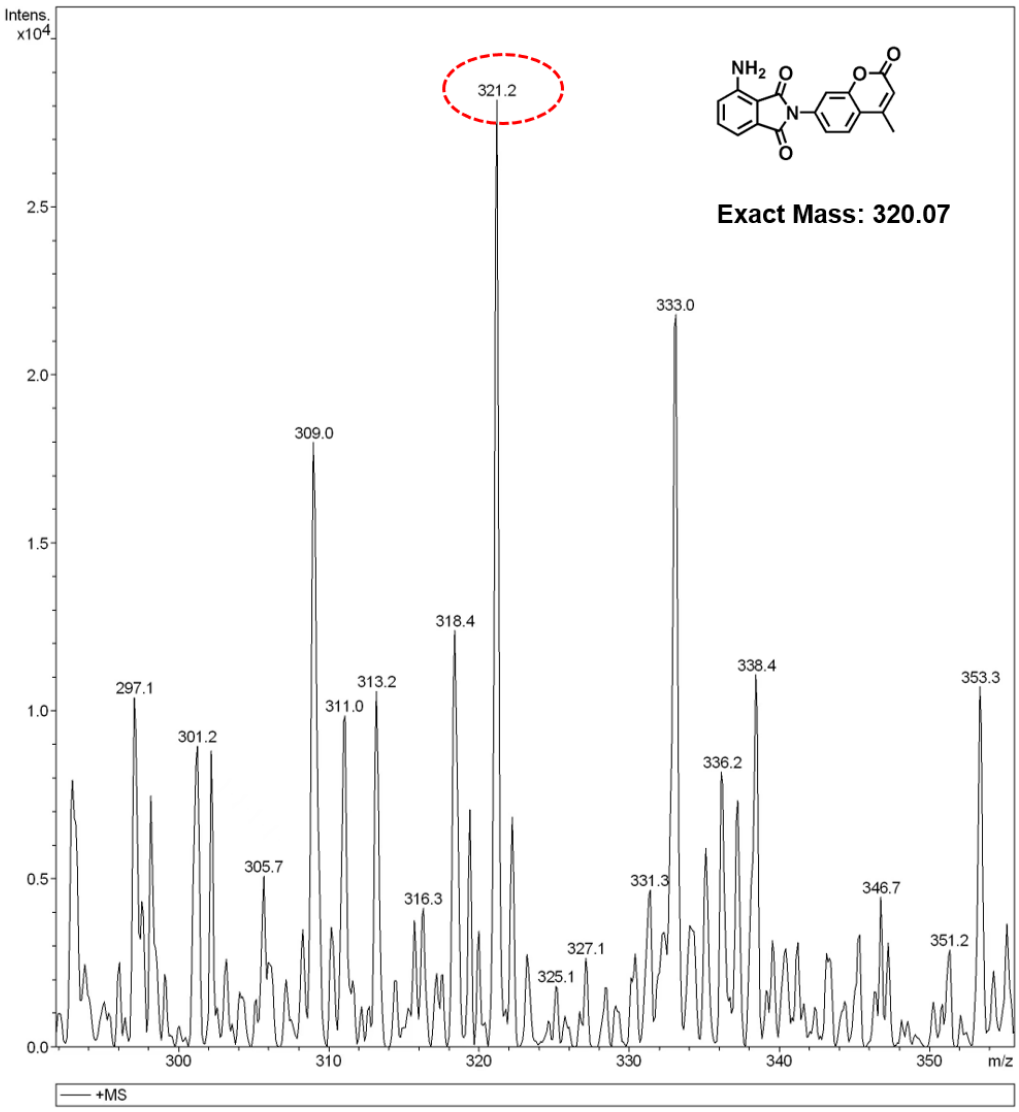

Figure S5 MS of the CF molecule.

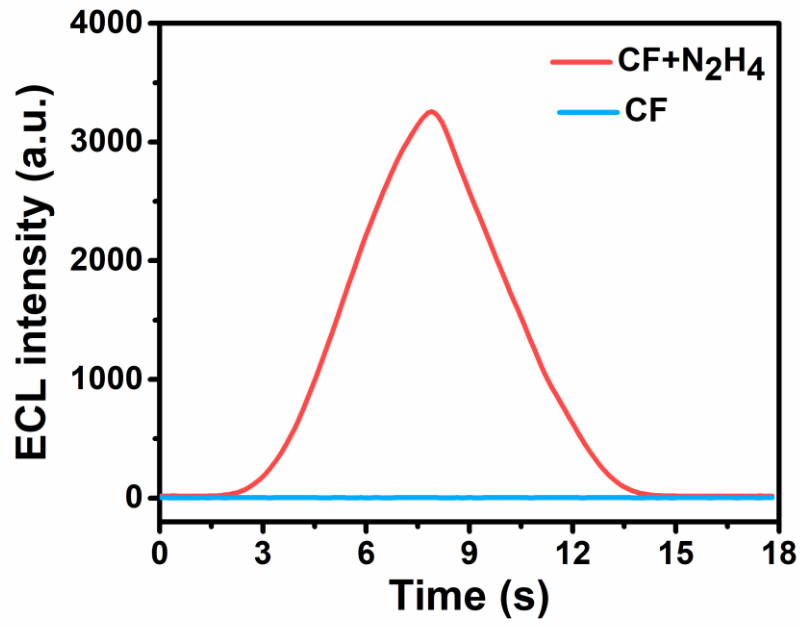

Figure S6 ECL response of CF to N_2H_4 at bare GCE in 10 mM PBS (pH = 7.4) containing 0.05 mM H_2O_2.

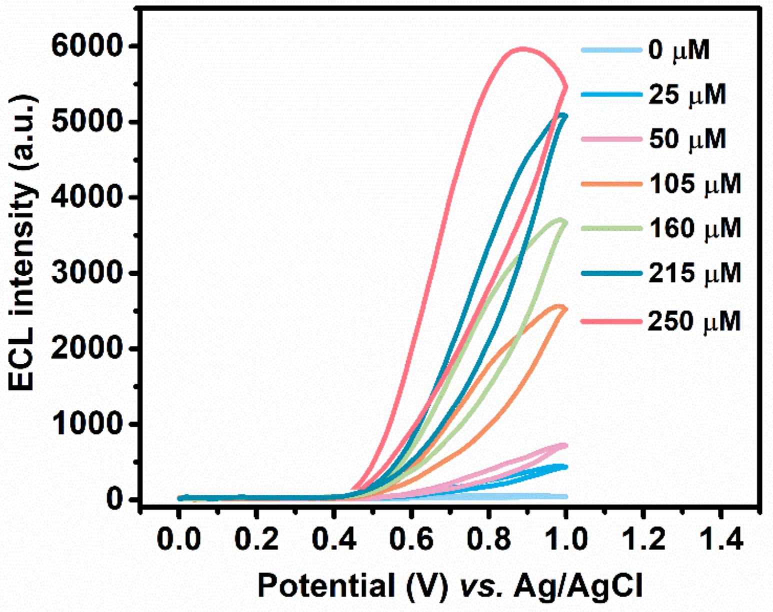

Figure S7 The relationship between ECL intensity and concentrations of N_2H_4 in the range of 0 – 250 μM.

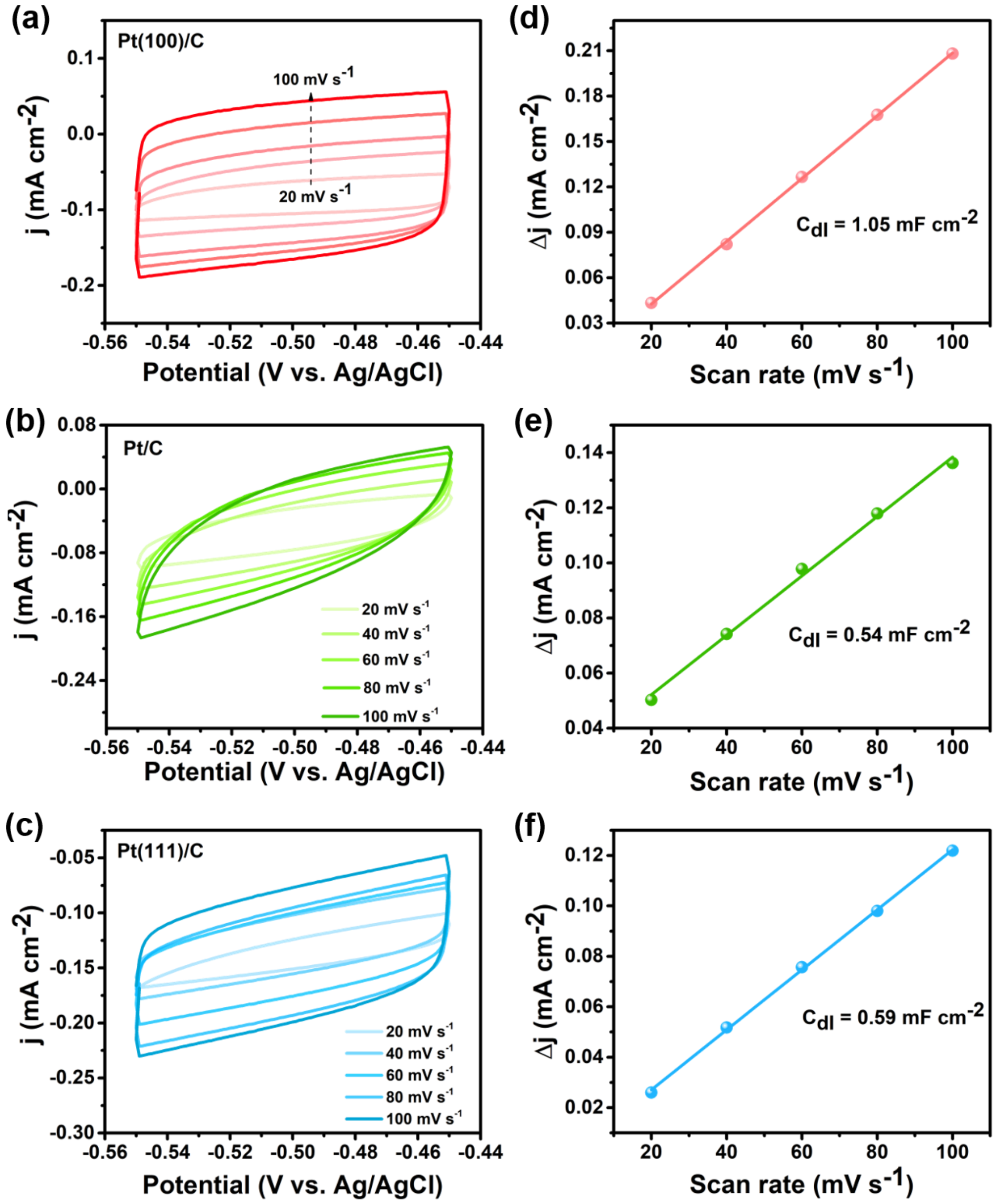

Figure S8 The electrochemical active surface area (ECSA) measurements on electrocatalysts. CV curves of (a) Pt(100)/C, (b) commercial Pt/C and (c) Pt(111)/C. (d-f) C_{dl} of corresponding electrocatalysts.

The electrochemical active surface area (ECSA) measurements of three Pt-based catalysts was estimated by measuring the capacitive current associated with double-layer charging from the scan-rate dependence of cyclic voltammograms (CVs). The potential window of CVs was - −0.55 V to −0.55 V (vs. Ag/AgCl) in a non-Faradic potential range under different scan rates with 20, 40, 60, 80 and 100 mV s^{-1}. The double layer capacitances (C_{dl}) of three Pt-based

catalysts were estimated by plotting the $\Delta J = J_a - Jc$ at −0.50 V (vs. Ag/AgCl) against the scan rate. The linear slope is equivalent to twice of the double-layer capacitance C_{dl}, and the ECSA of catalyst was calculated by the following equation: ECSA = C_{dl}/C_s. Where C_s is the specific capacitance of planar surface with an atomically smooth under identical electrolyte conditions, 60 μF cm^{-2} is used in this work. The ECSA of Pt(100)/C, commercial Pt/C and Pt(111)/C is 17.5, 9.0 and 9.8, respectively.

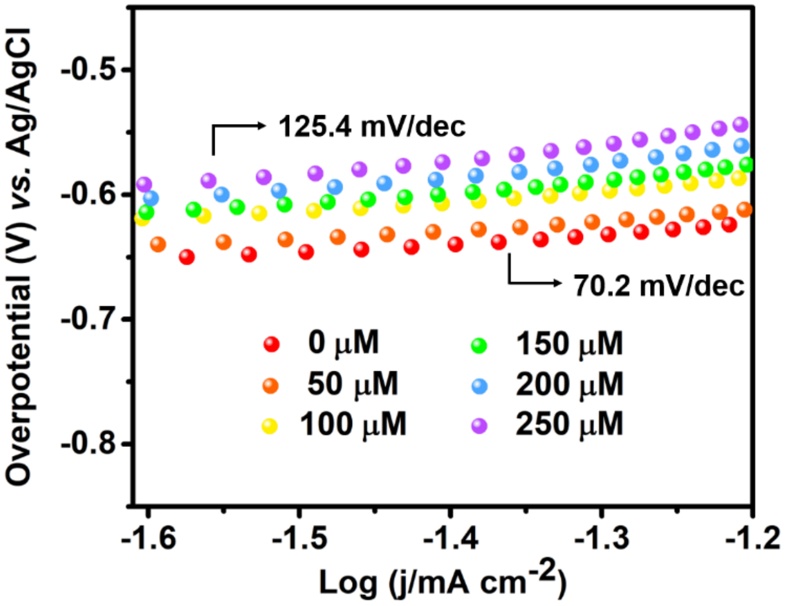

Figure S9 Tafel curve of commercial Pt/C catalysts. Tafel slope shows an increasing trend with the accumulation of CF concentration.

Tafel analysis is an effective electrochemical tool for studying the reaction pathways of electrocatalytic processes. Generally, the smaller the Tafel slope value, the faster the current density increases, indicating faster catalyst kinetics and better catalytic activity. The Tafel slope value was calculated according to according to the Tafel equation:

$$\eta = a + blogi \qquad (7)$$

where η is the AOR overpotential, b is the Tafel slope.

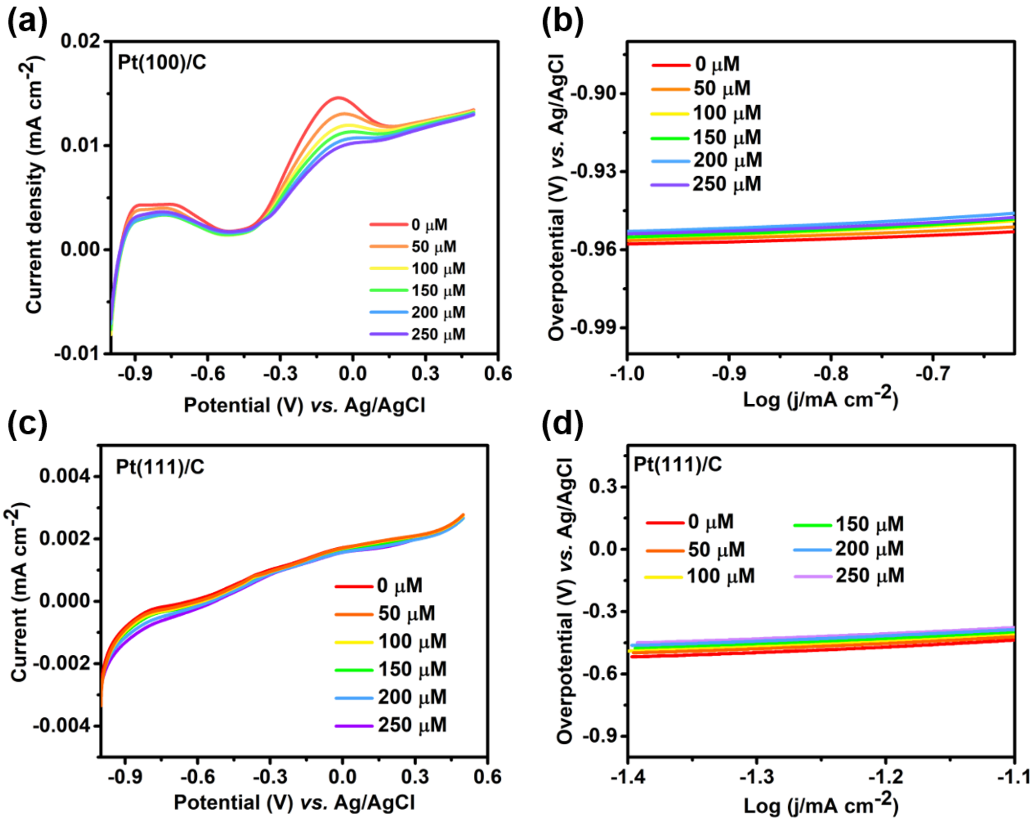

Figure S10 (a) Linear sweep voltammetry curves of the Pt(100)/C and (c) Pt(111)/C electrocatalyst in 0.1 M KOH with the titration of 0 − 250 μM probes. (b) The calculated Tafel slopes over Pt(100)/C and (d) Pt(111)/C with different titration of probes.

When the probe CF is not added, the calculated Tafel slope values of Pt(111)/C and Pt(100)/C catalyst are 281.3 mV/decade and 10.4 mV/decade, respectively.

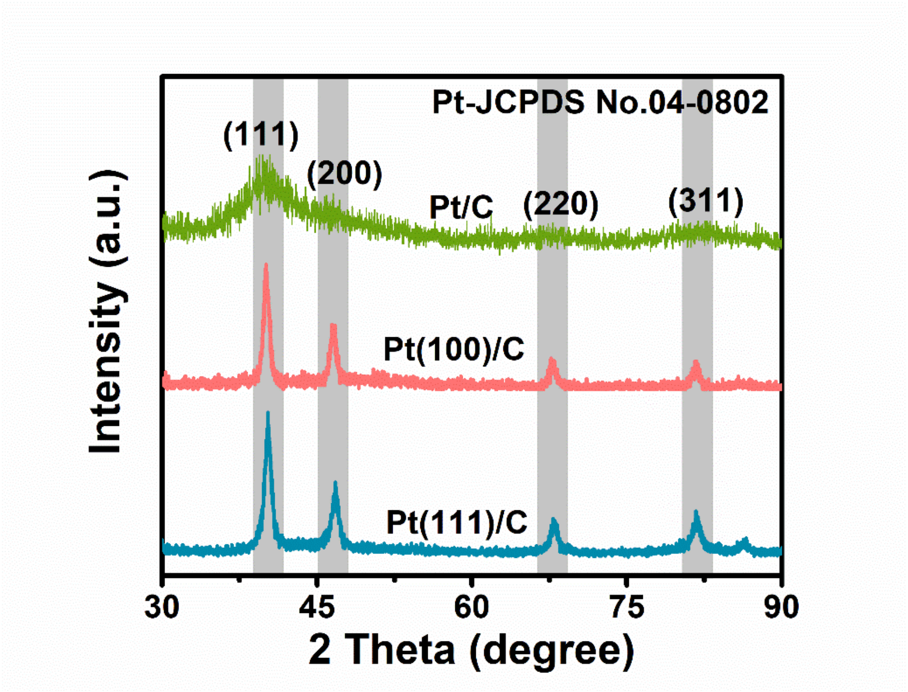

Figure S11 XRD patterns of commercial Pt/C, Pt(100)/C and Pt(111)/C.

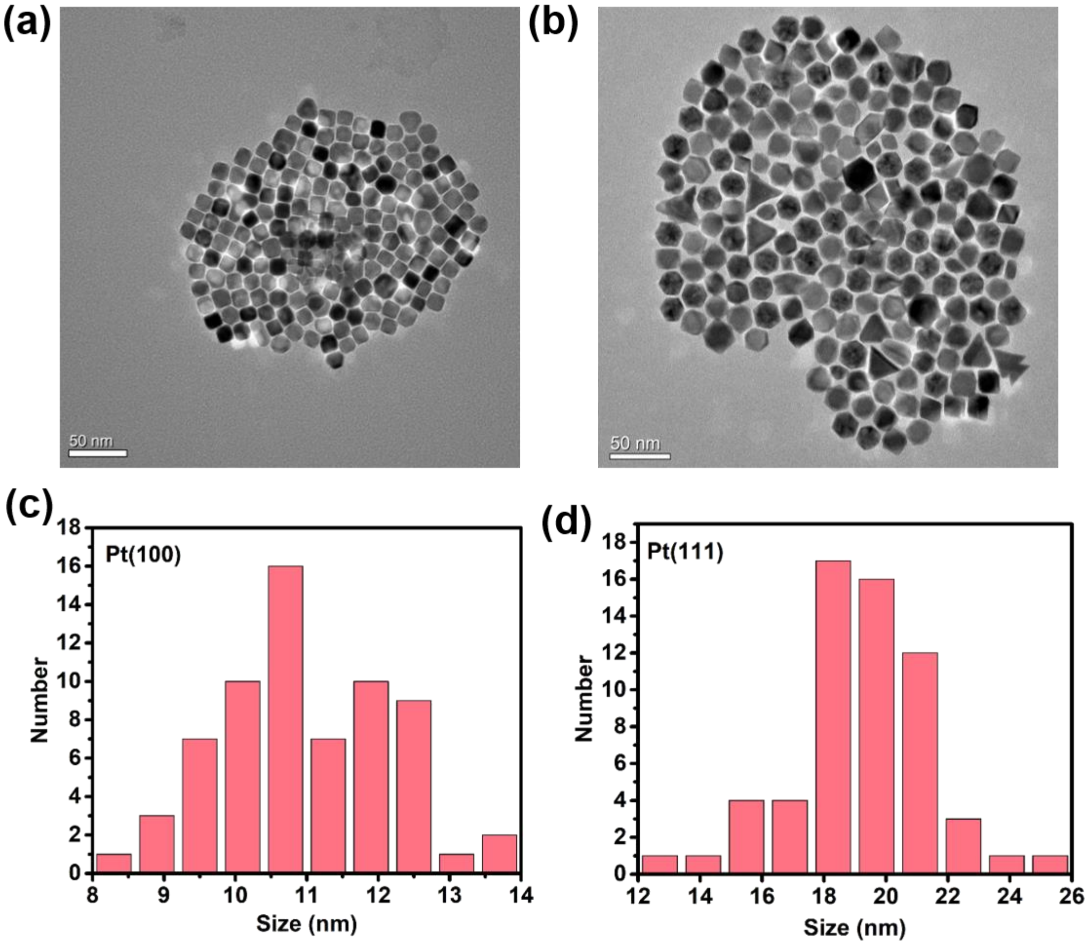

Figure S12 (a) TEM photographs of Pt(100)/C and (b) Pt(111)/C. (c) Dimension distribution drawings Pt(100)/C and (d) Pt(111)/C.

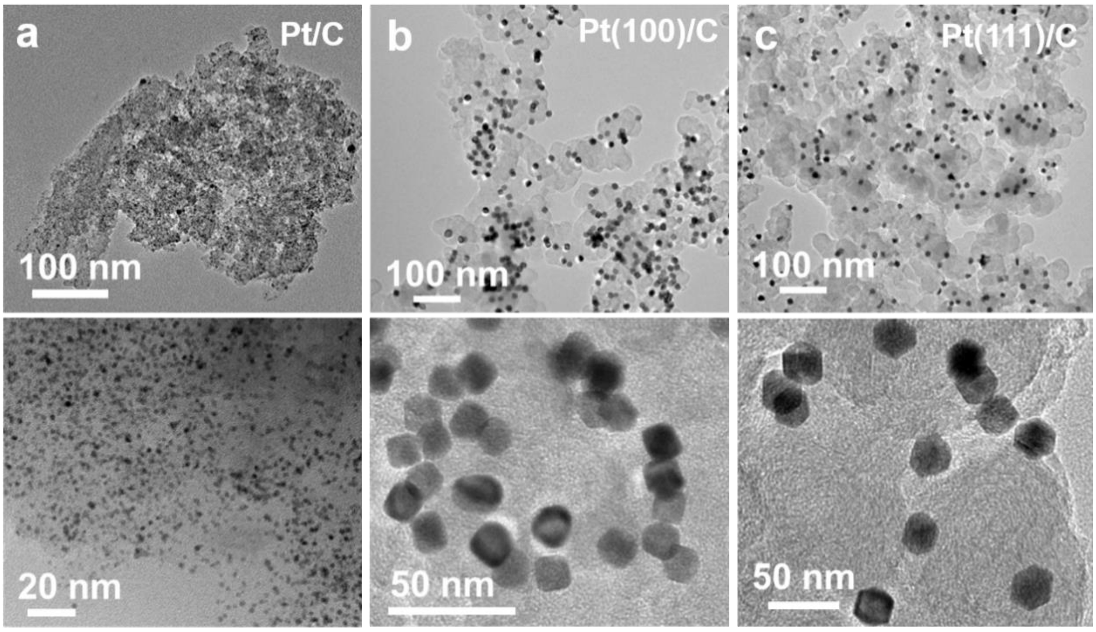

Figure S13 TEM photographs of (a) Pt/C, (b) Pt(100)/C and (c) Pt(111)/C.

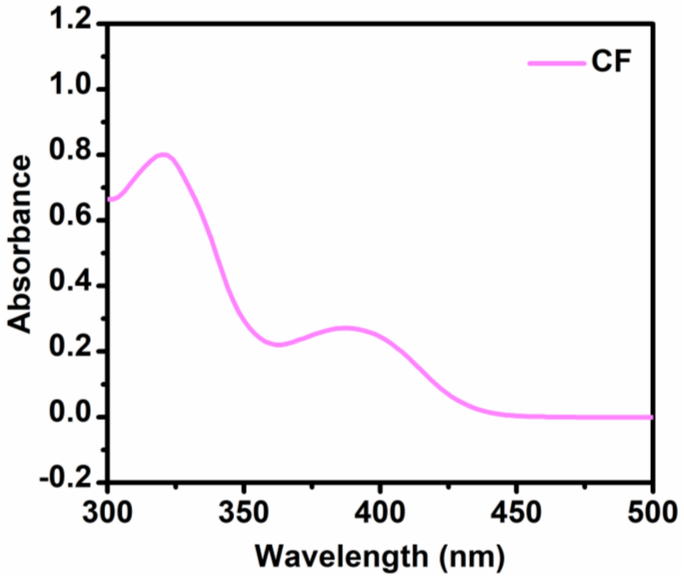

Figure S14 UV-visible spectra of CF in H_2O/DMSO (3:7, v/v).

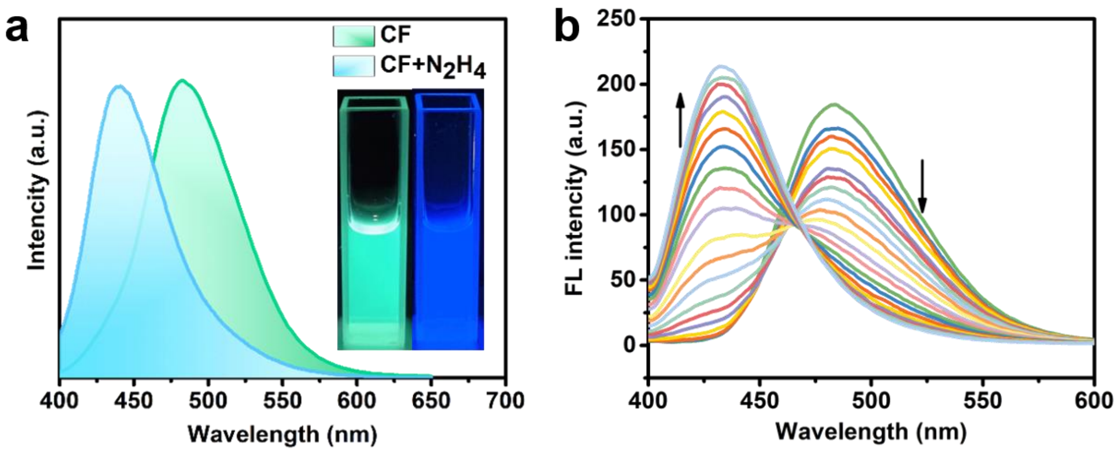

Figure S15 (a) Fluorescence spectra of probe CF (1.0 μM) recorded without and with hydrazine (1.1 μM) in an aqueous mixture (H_2O/DMSO: v/v, 3:7). Inset: the fluorescence color changes (from green to blue) and fluorescence imaging of CF before and after the reaction with N_2H_4. (b) Fluorescent spectra changes of CF upon addition of hydrazine (λ_{ex} = 380 nm). Each spectrum was recorded after reaction of the probe with the various species for 0.5 min in a mixture of H_2O/DMSO (3:7, v/v) at room temperature and obtained using excitation at 380 nm. CF: 2.2 μM in H_2O/DMSO (v/v, 3:7), N_2H_4 concentration: 0, 0.1, 0.2, 0.3, 0.4, 0.5, 0.6, 0.7, 0.8, 1.0, 1.1, 1.2, 1.3, 1.4, 1.6, 1.7, 1.8, 1.9, 2.0 μM.

With the increase of N_2H_4 concentration, the emission of CF at 480 nm diminished gradually, and a new emission peak was formed at 430 nm (λ_{ex} = 380 nm), which is associated with the released AMC. Additionally, the fluorescence color of the probe changed from green to blue after the reaction with N_2H_4.

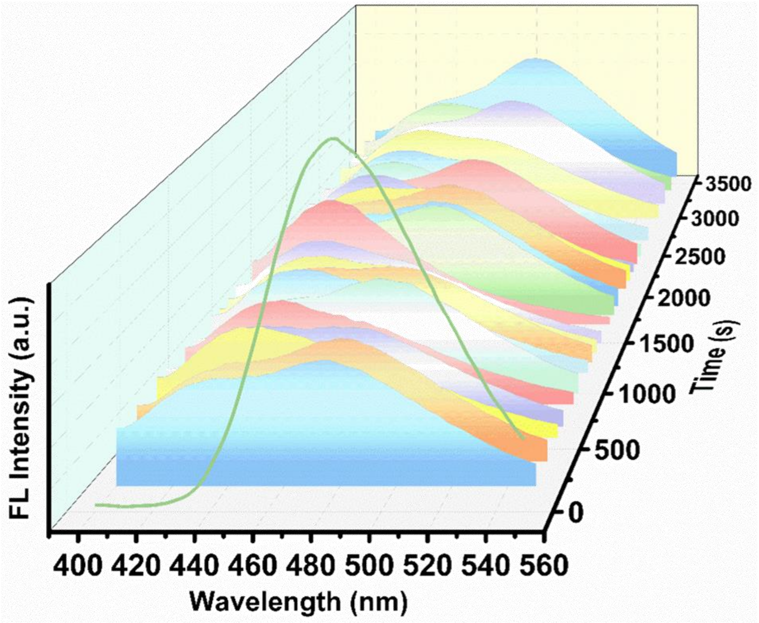

Figure S16 Time-dependent fluorescence spectra during electrochemical AOR process.

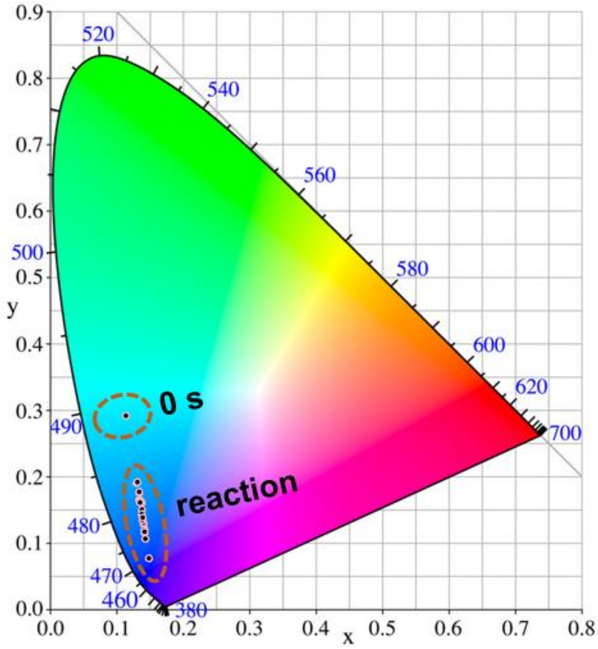

Figure S17 Corresponding CIE coordinate diagram during electrochemical AOR process (CF concentration: 2 μM).

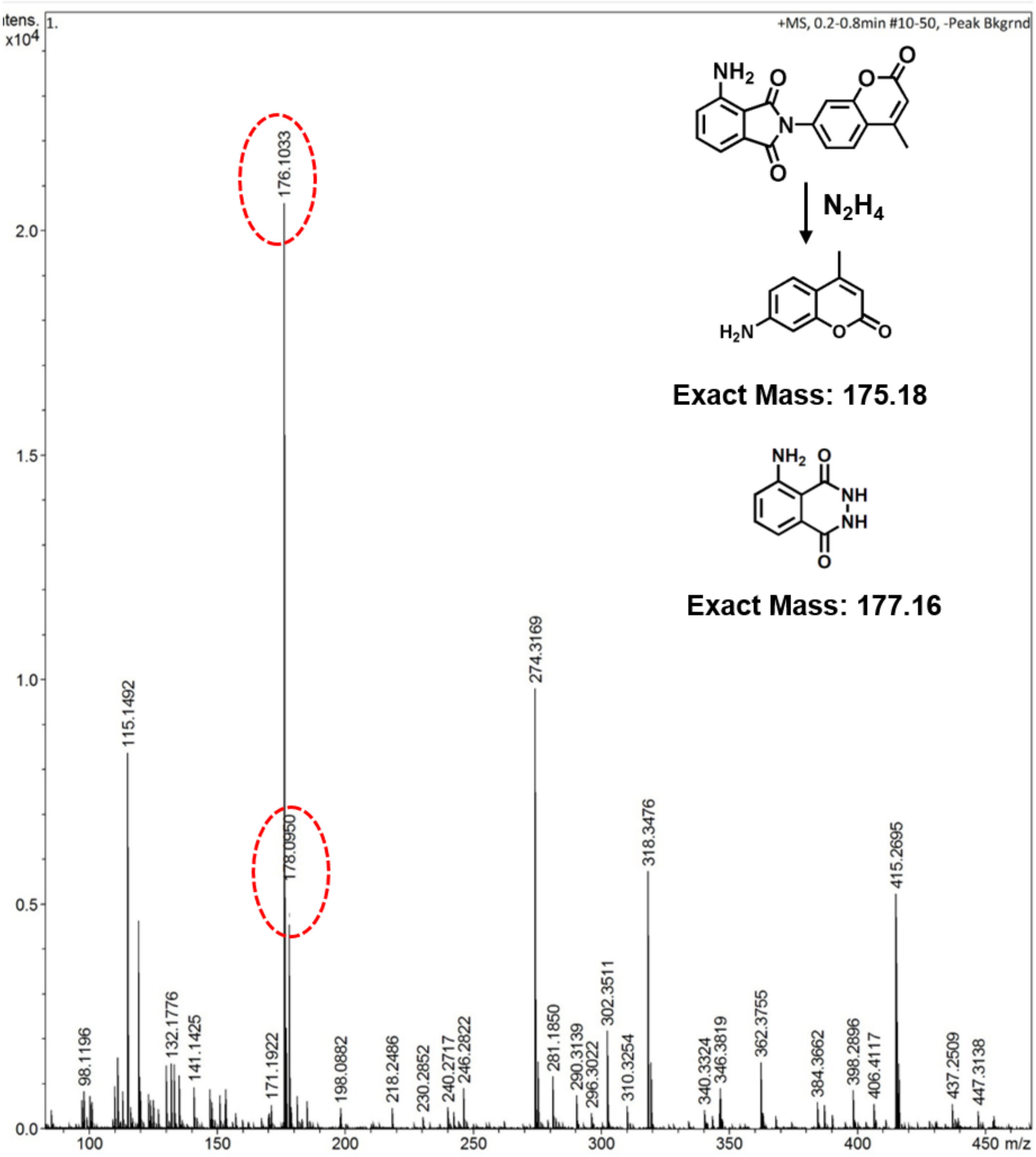

Figure S18 MS of the reaction product of CF with N_2H_4. The spectra fragments of m/z = 176.10 and 178.09 are attributed to the fragments $[AMC + H]^+$ and $[luminol + H]^+$, respectively.

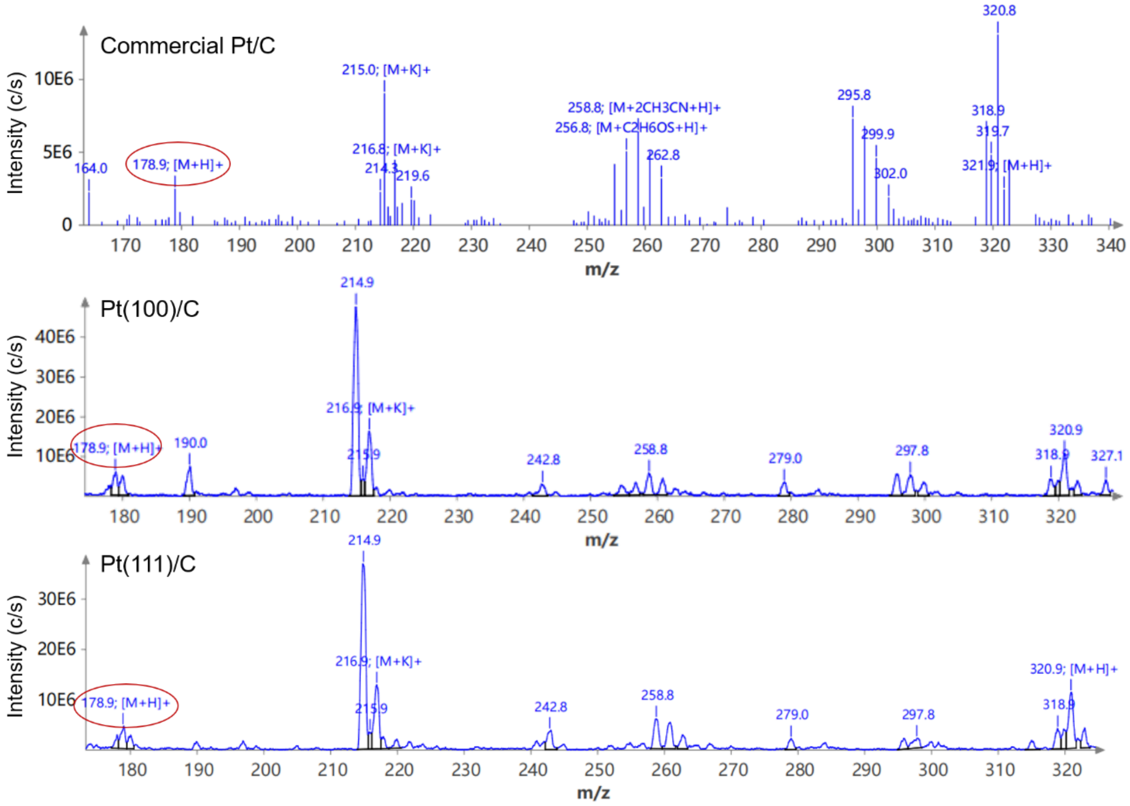

Figure S19 MS of the reaction product of CF with N_2H_4 intermediate after 20 min of AOR process on three Pt-based electrocatalysts. The spectra fragments of m/z = 178.9 are attributed to the fragments [luminol + H]$^+$.

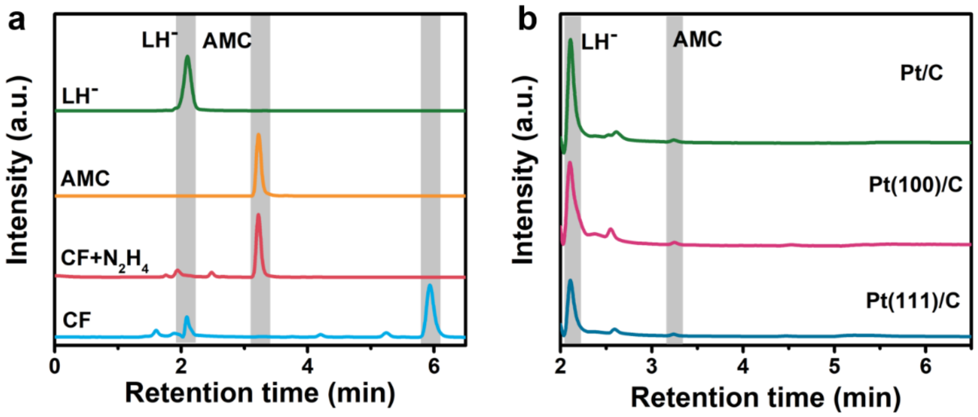

Figure S20 (a) HPLC spectra (detected by UV) of Lumino (LH^- under alkaline conditions), AMC, probe CF and product of CF after hydrazinolysis; (b) HPLC spectra of product of CF with N_2H_4 intermediate after 20 min of AOR process on three Pt-based electrocatalysts.

The reaction solutions were analyzed by HPLC after filtering through a filter (MILLEX@GP, 0.22 μm, Carrigtwohill, Co Cork). HPLC conditions were as follows: MeCN: H_2O=50:50 (v/v), flow rate 1 mL/min, detection wavelength of 356 nm. The reaction solution of CF (20 μM) and hydrazine (1 mM) which was incubated in PBS buffer (0.01 M PBS, pH =7.4, contains 10% DMSO) for 1 min. After reaction, the solution was filtered through filter. In the electrocatalytic experiment, 5mL of AOR reaction solution was reacted with CF (2 μM).

References

[1] G. Kresse, J. Furthmüller, *Comput. Mater. Sci.* **1996**, *6*, 15−50.

[2] G. Kresse, J. Furthmüller, *Phys. Rev. B* **1996**, *54*, 11169−11186.

[3] J. P. Perdew, K. Burke, M. Ernzerhof, *Phys. Rev. Lett.* **1996**, *77*, 3865−3868.

[4] G. Kresse, D. Joubert, *Phys. Rev. B* **1999**, *59*, 1758-1775.

[5] P. E. Blöchl, *Phys. Rev. B* **1994**, *50*, 17953−17979.
